# Objective Assessment of Cognitive Workload in Surgery: A Systematic Review

**DOI:** 10.1097/SLA.0000000000006370

**Published:** 2024-06-07

**Authors:** Aws Almukhtar, Virginia Caddick, Ravi Naik, Mary Goble, George Mylonas, Ara Darzi, Felipe Orihuela-Espina, Daniel R. Leff

**Affiliations:** * Department of Surgery and Cancer, Imperial College London, London, UK; † Department of Computer Science, University of Birmingham, Birmingham, UK; ‡ Department of Breast Surgery, Imperial College Healthcare NHS Trust, Charing Cross Hospital, London, UK

**Keywords:** cognitive workload, mental effort, patient safety, surgical skills, surgical training

## Abstract

**Objective:**

To systematically review technologies that objectively measure cognitive workload (CWL) in surgery, assessing their psychometric and methodological characteristics.

**Background:**

Surgical tasks involving concurrent clinical decision-making and the safe application of technical and non-technical skills require a substantial cognitive demand and resource utilization. Cognitive overload leads to impaired clinical decision-making and performance decline. Assessing CWL could enable interventions to alleviate burden and improve patient safety.

**Methods:**

Ovid MEDLINE, OVID Embase, the Cochrane Library, and IEEE Xplore databases were searched from inception to August 2023. Full-text, peer-reviewed original studies in a population of surgeons, anesthesiologists or interventional radiologists were considered, with no publication date constraints. Study population, task paradigm, stressor, cognitive load theory (CLT) domain, objective and subjective parameters, statistical analysis, and results were extracted. Studies were assessed for (1) definition of CWL; (2) details of the clinical task paradigm; and (3) objective CWL assessment tool. Assessment tools were evaluated using psychometric and methodological characteristics.

**Results:**

A total of 10,790 studies were identified; 9004 were screened; 269 full studies were assessed for eligibility, of which 67 met inclusion criteria. The most widely used assessment modalities were autonomic (32 eye studies and 24 cardiac). Intrinsic workload (eg, task complexity) and germane workload (effect of training or expertize) were the most prevalent designs investigated. CWL was not defined in 30 of 67 studies (44.8%). Sensitivity was greatest for neurophysiological instruments (100% EEG, 80% fNIRS); and across modalities accuracy increased with multisensor recordings. Specificity was limited to cardiac and ocular metrics, and was found to be suboptimal (50% and 66.67%). Cardiac sensors were the least intrusive, with 54.2% of studies conducted in naturalistic clinical environments (higher ecological validity).

**Conclusions:**

Physiological metrics provide an accessible, objective assessment of CWL, but dependence on autonomic function negates selectivity and diagnosticity. Neurophysiological measures demonstrate favorable sensitivity, directly measuring brain activation as a correlate of cognitive state. Lacking an objective gold standard at present, we recommend the concurrent use of multimodal objective sensors and subjective tools for cross-validation. A theoretical and technical framework for objective assessment of CWL is required to overcome the heterogeneity of methodological reporting, data processing, and analysis.

Estimators of cognitive workload load (CWL) have been found to be good predictors of psychomotor skill acquisition and task performance.^1,2^ As illustrated in Figure 1, multiple factors related to the task, the operator, and the operating environment affect CWL, and, by extension, surgical performance.^3–5^ At the operator level, increased CWL, brought on by short-term mental, emotional, or physical factors, or by long-term fatigue and burnout, which have increased significantly following the COVID-19 pandemic,^6^ may have a detrimental effect on performance, patient safety, the health care system, and the broader economy.^4,7–10^ Up to one third of surgical errors can be attributed to fatigue and excessive workload^11^; burnout is significantly associated with surgical errors.^12^ In a cognitively demanding environment, such as operating rooms, both high CWL states (cognitive overload), and inattention and environmental distraction of low CWL states (cognitive underload), can lead to errors and performance decline.^13–17^

**FIGURE 1:**
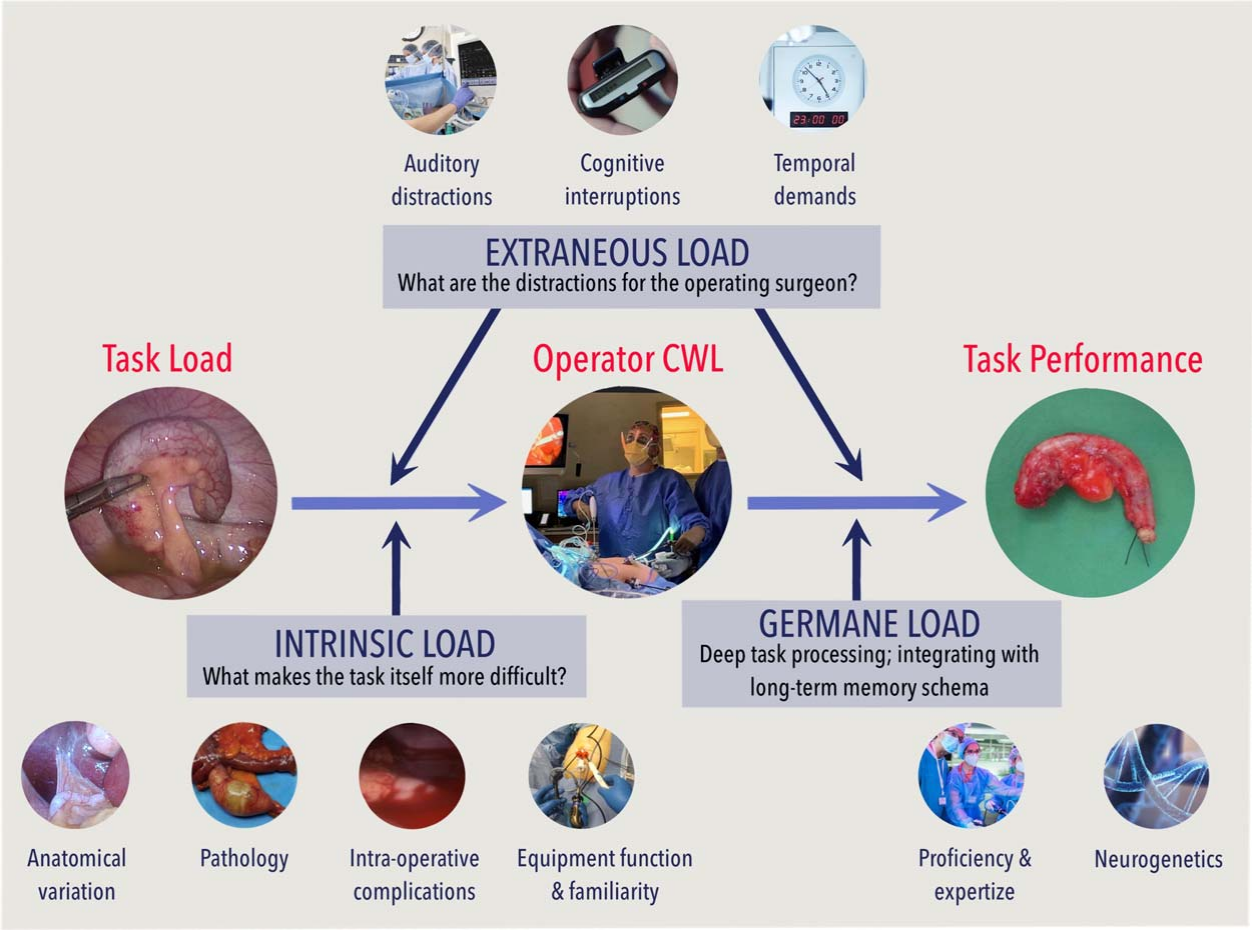
Sources of CWL during surgical task performance. Establishing CLT domain of a clinical procedure and study task paradigms: (a) INTRINSIC LOAD: the complexity of the task itself; (b) EXTRANEOUS LOAD: distractions for the operator; (c) GERMANE LOAD: the schema developed from previous processing to assist in task completion. CWL characterizes the relationship between task demands and the operator’s finite information-processing resources; with increasing task difficulty, cognitive resources are depleted, and CWL increases. CWL indicates cognitive workload.

Historically, CWL assessment was limited to subjective methods.^18^ These retrospective self-report measures may introduce granularity, domain-specific constraints, and recall bias.^19,20^ By contrast, physiological (autonomic or neuroimaging) assessment of CWL offers the potential for objective assessment of workload in near real-time and to detect changes in CWL as it occurs during an operation.^21–24^ As illustrated in Figure 2, these systems may improve surgical performance and patient outcomes by minimizing human error and ameliorating high workload. Prior systematic reviews in this area have failed to distinguish between “physiological stress” and CWL in their design^21,24^ and have limited scope.^23,25,26^ Furthermore, no study has attempted to formally evaluate the diagnostic accuracy of each modality by comparing sensitivity and specificity. Supplemental Digital Content 1, Table 1 (http://links.lww.com/SLA/F133) outlines key definitions and explanations of the main CWL objective assessment modalities as well as the Eggemeier criteria.

**FIGURE 2:**
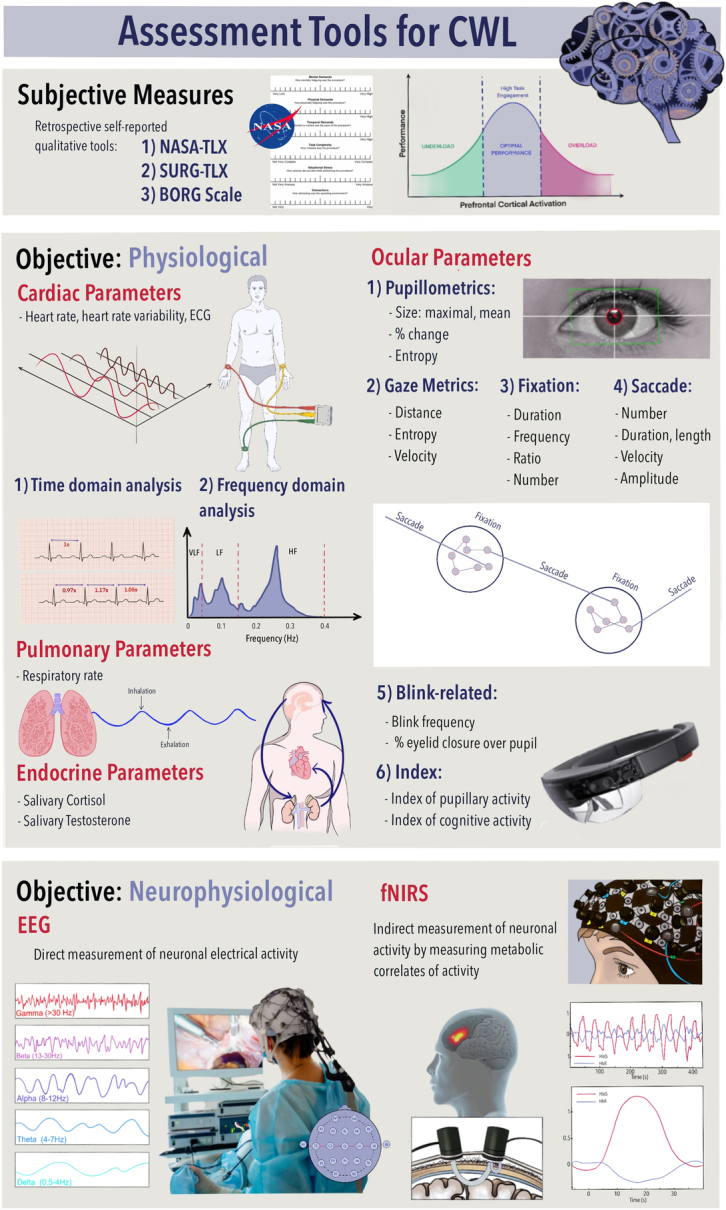
Objective and subjective methods used for CWL assessment. Different modalities are utilized in the attempts to assess CWL objectively. These can be categorized into autonomic metrics and neurophysiological metrics. In the surgical literature, the autonomic response to discernible changes in CWL can be detected by various cardiac (captured with ECG) and ocular metrics (captured with eye trackers), while the neurophysiological changes can be detected using EEG and fNIRS—modalities which detect neurological activity within a brain region. Please see Table 1 (Supplemental Digital Content 1, http://links.lww.com/SLA/F133) for an outline of key definitions. ECG indicates electrocardiography; EEG, electroencephalography; fNIRS, functional near-infrared spectroscopy; NASA-TLX, The National Aeronautics and Space Administration Task Load Index; SURG-TLX, The Surgery Task Load Index.

## METHODS

### Study Design

We conducted a systematic review of randomized and nonrandomized CWL assessment modalities in surgery, anesthesiology, and interventional radiology. Registered with “International Prospective Register of Systematic Reviews” (PROSPERO; CRD42023358935), the review was undertaken in accordance with the Cochrane Collaboration Recommendations and reported in line with the Preferred Reporting Items for Systematic Reviews and Meta-analyses (PRISMA) reporting guidelines.^27^

### Data Sources

A search of Ovid MEDLINE, Ovid Embase, the Cochrane Library, and IEEE Xplore databases was undertaken from inception until August 2023.

### Search Strategy

Medical Subject Heading (MeSH) and non-MeSH search terms encompassing “cognitive workload,” “objective measurement,” and “surgeons” were combined using Boolean string logic. All studies relating to tasks within surgery, interventional radiology, and anesthesiology were considered for completeness. Supplemental Digital Content 1, Table 2 (http://links.lww.com/SLA/F133) outlines the the full search terms.

### Selection Process

Articles were uploaded onto Covidence (Veritas Health Innovation) (available at www.covidence.org) and subjected to title and abstract screening by 2 of 3 reviewers (A.A., R.N., and V.C.) independently. Inclusion criteria included any study published in English, investigating the appropriate specialty population, all study designs reporting an objective measure of CWL detection, and ensuring alteration of mental demand within the task paradigm. Conversely, if the study did not investigate surgeons, anesthesiologists, or interventionalists, or did not test and report objective CWL measurement, it was excluded. Full inclusion and exclusion criteria are outlined in Table 3 of the Supplemental Digital Content 1 (http://links.lww.com/SLA/F133).

### Data Collection Process and Quality Assessment

Data were extracted using an agreed data extraction template outlined in Table 4 of the Supplemental Digital Content 1 (http://links.lww.com/SLA/F133). A Newcastle-Ottawa scale (NOS),^28^ was used to assess the methodological quality of studies (see supplemental content for both, Supplemental Digital Content 1, http://links.lww.com/SLA/F133). The NOS assesses the quality of nonrandomized studies and has nonrandomized and content validation for use in meta-analysis. Full-text review, data extraction, and quality assessment were undertaken by 2 of 4 reviewers (A.A., R.N., M.G., and V.C.) independently; disagreements were resolved by consensus and in discussion with senior authors (F.O.-E. and D.R.L.) where required.

### Primary Outcome

The primary outcome was to evaluate the diagnostic accuracy of different methods for CWL assessment in surgery. Diagnostic accuracy of each modality was inferred by calculating the sensitivity (calculated by dividing the number of true positives by the sum of true positives and false negatives) and specificity (true negative/true negative+false positive). True and false negatives were categorized based on the following assumptions: (1) the task paradigm is designed to result in change in CWL (one of our inclusion criteria) and (2) the reported subjective measure is gold standard.

### Secondary Outcomes

The secondary outcomes were to identify an agreed definition for CWL, to identify the modalities used to objectively assess CWL in surgical tasks, and to compare the modalities according to the Eggemeier criteria—that is, sensitivity, selectivity, diagnosticity, reliability, intrusiveness, implementation requirements, and operator acceptance.^29^

## RESULTS

### Study Selection

**FIGURE 3:**
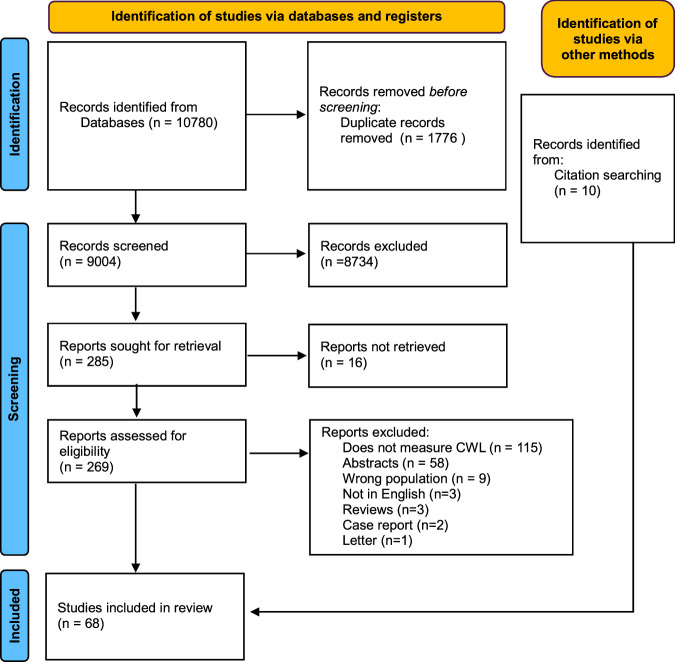
PRISMA flowchart outlining study identification means, screening process, and included and excluded studies.

A PRISMA flow diagram illustrating the systematic review process is provided in Figure 3. The initial searches yielded 10,780 studies. In all, 1776 articles were excluded as duplicates; a further 8784 were excluded following screening of abstracts. Of the 285 remaining, 16 were unable to be retrieved, leaving 269 studies which were subjected to full-text screening, of which 58 studies met the inclusion criteria. A further 10 studies were identified upon bibliographic cross-referencing, resulting in 68 studies for final systematic review. One study, Dalveren et al 2018 (ii)^30^ was a retrospective analysis of a previously published experiment Dalveren and Cagiltay 2018 (i)^31^ and included more parameters; thus, was counted as one study in the analysis. Heterogeneity in task paradigms, objective metrics, and reporting of results precluded a quantitative meta-analysis. Overall, 56 studies (13.4%) were deemed good quality^31–85^; 9 fair^86–94^; and 2 poor^95,96^ (Supplemental Digital Content 1, Table 5, http://links.lww.com/SLA/F133). Tables 6–8 in the Supplementary Digital Content 1 (http://links.lww.com/SLA/F133) summarize a description of each paper including year of publication, setting, sample number, study design.

### Primary and Secondary Outcomes

Of the 67 studies, 14 (20.9%) clearly defined CWL^35,42,54,60,61,66,69,71,73,76,80,82,90,92^; 23 (32.3%) referred to “workload principles”^31,37,39,40,50,51,59,62–65,68,72,75,77,81,84,85,87,93,94,96,97^; and 30 studies (44.8%) made no attempt to define CWL.^32–34,36,38,41,43–49,52,53,55–58,67,70,74,78,79,86,88,89,91,95,98^ Nine studies (13.4%) used CWL interchangeably with “stress.”^32–34,36,38,41,43–49,52,53,55–58,67,70,74,78,79,86,88,89,91,95,98^

Thirty-nine studies (58.2%)^32–35,37,38,41,43–46,49,55,57,61–65,67,69,73,74,76–82,85–88,90,92–94,96^ used 1 or more subjective CWL assessment tools. NASA-TLX was the most widely reported (26 studies);^32,34,38,41,43,44,46,49,61,65,69,74,77–82,85–88,90,92,93,96^ 11 used SURG-TLX^33,35,45,55,57,62–64,67,73,94^; 3 used the Borg Scale^44,61,76^; and 1 Likert Scale of perceived workload.^37^ Table 9 (Supplemental Digital Content 1, http://links.lww.com/SLA/F133) summarizes the main study characteristics by modality.

### Objective Measurement of CWL

For each modality (eg, ocular), we identified multiple parameters (eg, gaze, pupillometry, saccade) and numerous metrics within each respective parameter (eg, saccade number, duration, velocity, amplitude, and length). Seven parameters were identified within 33 ocular studies. These included pupillometrics,^31,35,38,39,42,47–51,54,57,68,70,71,74,77,78,82,84,91^ fixations,^31,34,36,38,47–51,57,70,71,78,79,82,83^ gaze,^36,43,44,46,77,78^ saccades,^36,38,47,50,51,70,82,98^ blinks,^34,35,37,47,48,57,68,78,82,85^ area of interest (AOI),^36,38,48,74,79^ and indices.^50,51,68^ Table 10 (Supplemental Digital Content 1, http://links.lww.com/SLA/F133) illustrates the diversity of individual metrics reported. The 24 cardiac studies^32,33,40,41,45,55,56,61–64,67,69–71,73,76,80,81,86,87,89,94,95^ were similarly diverse. Two studies^33,41^ employed hormonal assays, with conflicting findings on the detection of changes in CWL, while 3 studies^55,69,86^ utilized respiratory parameters, but only 1 illustrated an association between changes in respiratory rate and CWL. Consequently, respiratory and hormonal metrics were excluded from further analysis.

For neurophysiological metrics, a variety of hardware, configuration (channel, electrode numbers, and montage setups), and areas of interest were studied^1,4,64,65,93^; electrodes were used for EEG and 16 to 40 optodes for fNIRS.^53,75^ All fNIRS studies reported change in oxygenated hemoglobin (ΔHbO_2_), 6 studies^53,58,62,64,73,97^ also reported changes in deoxygenated hemoglobin (ΔHHb)^53,58,62,64,73,97^ and 1 calculated “OXY” (ΔHbO_2_−ΔHHb).^53^ Analysis methods were the sole source of study heterogeneity for fNIRS. Certain investigators chose to pool data across the entire prefrontal cortex (PFC)^41,58^; others reported on individual channels,^53,58,62–64,73,75^ or by anatomical region of interest (ROI).

### Formal Methodological and Psychometric Evaluation: Comparison of Metrics as CWL Assessment Tools

Deductive analysis was carried out against a methodological framework^29^ for evaluating CWL tools: the sensitivity, selectivity (ie, specificity), diagnosticity, and reliability in reference to instrument validity; while intrusiveness, implementation, and acceptability were used to assess pragmatic utility. Table 11 (Supplemental Digital Content 1, http://links.lww.com/SLA/F133) illustrates the inductive analysis of task paradigm to determine (1) which CLT domain (ie, extraneous, intrinsic, germane, or uncontrolled) is responsible for the change in CWL and (2) to determine whether the modality had detected the change in CWL

#### Sensitivity

Table 12 (Supplemental Digital Content 1, http://links.lww.com/SLA/F133) summarizes the sensitivity of each modality (with and without the inclusion of multimodal studies), comparing against a “gold-standard” subjective measure (NASA-TLX, SURG-TLX, and Borg Scale). Neurophysiological metrics were observed to have superior sensitivity compared with peripheral autonomic measurements. EEG and fNIRS demonstrated 100% and 80% sensitivity, respectively, in detecting the change in CWL compared with 90% and 76.2% in ocular and cardiac sensors, respectively.

When >1 objective modality was used (multimodal studies), sensitivity increased. Four fNIRS studies also reported cardiac metrics^41,62,64,73^ and 1 reported EEG metrics.^75^ When taking into account HF, LF, and mean HR, the sensitivity of fNIRS for detection increased to 87.5%.^41^ Similarly, the sensitivity of cardiac metrics increased to 85.1% when taking into account fNIRS data^64,73^ and EEG data.^80^ The sensitivity of ocular metrics increased to 92.86% when factoring in mean HR and HF/(HF+LF).^81^

Although EEG sensitivity was 100%, in the majority of studies only a single metric was found to be significant. Of the 14 studies, 1 found a single band frequency (beta) to be significant^65^; 3 multiple band frequencies (theta, beta, and alpha)^72,79,93^; 7 composite scores^52,59,77,80,88,90,96^; and 3 machine learning algorithms.^60,75,92^ All 9 fNIRS studies reported significant changes in ΔHbO_2_ with increased task CWL; 8 of the 9 fNIRS studies^53,62–64,73,75,97^ were significant on post hoc analysis, with the exception of Crewther et al.^41^ More specifically, analysis by anatomical subregion identified workload-related activations across the bilateral DLPFC and VLPFC.^53,62,63,73,75^

With regards to peripheral autonomic sensors, time domain HRV was most sensitive to changes in workload. PNN50^61,67,87^ and SDNN^61,67,87,95^ are sensitive to changes in CWL across all experiments, demonstrating 100% sensitivity. Conversely, the frequency domain metric, LF/HF ratio, used in 7 experiments, had a sensitivity of only 50%.^32,41,61,67,87,89,95^ Mean heart rate was most widely employed (69.23% sensitivity); however, methods of computation varied significantly.^32,38,40,41,55,56,61,70,71,73,76,80,81,87,89,94^ Ocular sensors revealed excellent sensitivity and were the most widely used metrics. Dwell time, saccade number, gaze velocity, and gaze entropy all showed 100% sensitivity.

#### Selectivity (ie, Specificity)

The ability to detect changes in cognitive demand, as opposed to other variables (eg, stress or physical workload) is paramount. The scarcity of false negatives meant that it was only possible to calculate the specificity for peripheral autonomic studies. For cardiac metrics, the specificity was only 50%; for ocular metrics, it was 66.7%.

#### Diagnosticity

**FIGURE 4:**
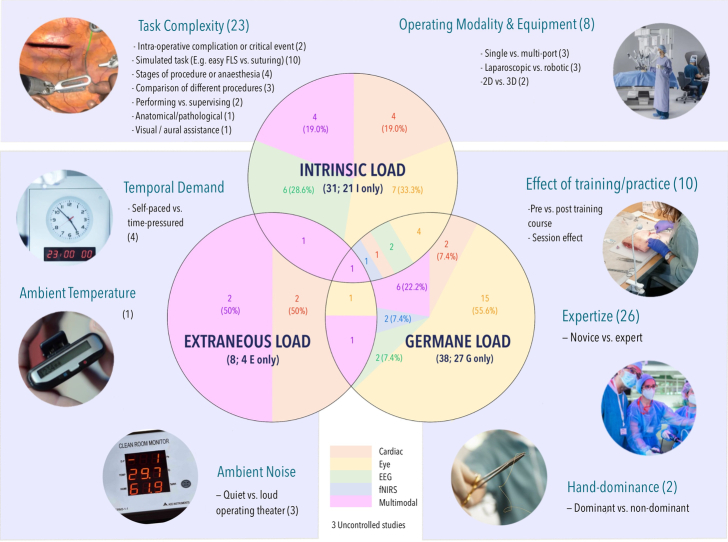
Venn diagram illustrating the distribution of studies by CLT domain, task paradigm, and modality. For each CLT domain, the pie chart demonstrates the proportion of sensor modality use (orange for cardiac, yellow for eye, green for EEG, blue for fNIRS, and pink for multimodal). Such breadth of study designs prevented meaningful quantitative repeatability analysis due to the paucity of studies investigating the same CLT domain, task paradigm and sensor, and employing comparable data analysis techniques.

Figure 4 and Table 11 (Supplemental Digital Content 1, http://links.lww.com/SLA/F133) illustrate the distribution of the CLT domain and task paradigm. The majority investigated only a single domain: 21 intrinsic,^33,43,46,52,54,55,61,65,67,70,72,79,82–84,88–92,95,96^ 4 extraneous,^56,62,63,94^ and 27 germane.^32,35,36,38–42,47,48,50,51,57,58,60,66,68,71,74,75,77,78,80,85,87,93,97^ Three real-life studies^45,69,86^ were classified as “uncontrolled” CLT domain: perfusionists performing cardiopulmonary bypass,^45^ thoracic surgeries comparing open versus robotic-assisted,^86^ and continuous recording throughout an operating list.^69^ Studies investigating multiple CLT domains concurrently illustrated an ability to differentiate task load source or “diagnosticity”: intrinsic and extraneous,^73^ intrinsic and germane,^31,34,37,44,52,53,59,76^ and extraneous and germane.^49,64^ Only one studied all 3 domains.^81^ Five of 12 studies^44,52,64,73,76^ analyzed the separate domains individually. Of these, only 3^52,73,76^ reported statistically significant objective findings for both domains studied: 1 multimodal study^73^ utilizing fNIRS and ∆HR; 1 EEG study^52^; and 1 cardiac (HR).^76^

#### Reliability

Inferences regarding repeatability can be made by examining studies investigating the same CLT domain and task paradigm with the same metric (and ideally a similar device). For extraneous load, the effect of temporal demand on laparoscopic suturing was the only example. For cardiac data, mean HR results were conflicting,^64,73^ whereas 3 of 4 fNIRS studies (75%)^62,63,73^ demonstrated similar time pressure effects upon ∆HbO_2_ (in line with subjective workload). Regarding intrinsic domain, although 8 studied operating modality or equipment,^33,44,55,65,72,73,89,98^ no studies used comparable task paradigms or sensors. Task complexity was more widely studied. Two studies^88,90^ used EEG during urological robotic procedures (ie, lymph node dissection vs UVA), with both using ABM X1 devices and correlated composite EEG scores with NASA-TLX domains. Results were contradictory: the former found no significant correlation for extended lymph node dissection, whereas the latter did (*r*=−0.74, *P*=0.05). Conversely, for UVA, Guru et al^88^ reported a correlation between EEG “workload” and NASA-TLX [mental demand (*r*=−0.53; *P*=0.02)], temporal demand (*r*=−0.56; *P*=0.01), performance (−0.46; *P*=0.05), and frustration (*r*=−0.48; *P*=0.04); whereas Hussein et al^90^ did not. Five studies^43,54,82,84,91^ utilized ocular metrics to compare increasing complexity of easy versus complex FLS tasks. Four studies^54,82,84,91^ used pupillometrics: 2 average rate of pupil diameter change^82,91^; 1 pupil diameter change over a 7-second window^54^; and 1 mean pupil diameter.^84^ All demonstrated significant changes in ocular metrics in line with increasing workload. No repeatability data was available for gaze entropy or velocity.^43^ For cardiac metrics, 2 studies evaluated the stage of anesthesia.^61,76^ Both studies found an increase in mean HR during the induction phase compared with the maintenance phase (*P*<0.05).

Of the 26 studies examining the germane effect of expertize, no truly comparable repeatability data could be obtained, primarily owing to differing submetrics and analytic approaches used. Two studies^64,75^ examined the effect of expertize upon intracorporal knot-tying with fNIRS; however, data analysis techniques varied. Modi et al^64^ found significantly greater (*P*<0.05) ∆HbO_2_ in the right DMPFC in self-paced suturing (channel 23) and B/LPFC when suturing under a time-pressurized condition (channels 1, 11, and 19) in experts compared with juniors or intermediate residents. Walia et al^75^ utilized a GLM analysis to identify error-related processes. Experts demonstrated left DLPFC/frontal ∆HbO_2_ in keeping with activation, together with global suppression of sensorimotor areas. Conversely, novices displayed widespread frontoparietal and sensorimotor error-driven activation.

#### Intrusiveness

No definitive intrusiveness data were identified. Inferences regarding interference with the primary task can be made by examining the task paradigm setting: 46 were simulated^31,32,34–39,41–44,46,47,49,52–54,57–60,62–66,68,70,71,73,75,77–85,87,88,91,93,96,97^ and 21 studies^33,40,45,48,50–52,55,56,61,67,69,72,74,76,86,88–90,92,94,95^ were conducted in real-life clinical environments. Assessment of changes in cardiac physiology was most commonly employed in real-life studies and used in just over half of all reports [13/24 (54.2%)].^33,40,45,55,56,61,67,69,76,86,89,94,95^ The only neurophysiological modality used in the operating room was EEG [4 of 14 studies (28.6%)].^72,88,90,92^ Despite the ease of wearability of eye trackers, only 4 of 32 (12.5%) were trialed in real-life studies.^48,50,51,74^

#### Implementation Requirements

Device setup, pretest calibrations, and sampling frequency were poorly reported, and analytic techniques varied substantially. For the most used cardiac metric (mean HR), Kennedy-Mets et al^56^ divided the continuous procedure recording into 18 five-minute segments, while Martin et al^61^ analyzed the data according to the stage of the procedure. Three of 14 (21.4%) EEG studies did not report any information regarding the band frequencies used or data units reported within results tables, or cite the processing algorithms applied. By comparison, the 9 fNIRS studies provided detailed information regarding montage setup, correction/filtering techniques employed, and algorithms used.

#### Operator Acceptance

Operator perception of the validity and overall usefulness can be inferred from utility. Across the 32 studies identified, eye metrics were the most studied, followed by cardiac (24), EEG (14), and finally fNIRS (9).

## DISCUSSION

This systematic review sought to assess the diagnostic accuracy of various modalities for measuring CWL objectively, and to establish a consensus on CWL definition to mitigate confusion with terms such as “stress”—a broader physiological and psychological response to perceived challenges. Analyzing the literature through a robust theoretical framework^29^ and employing a meticulous methodological approach for quantitative modality comparison revealed superior sensitivity of neurophysiological metrics compared with autonomic metrics in detecting changes in CWL.

In the literature, CWL has been defined as “the level of overall mental effort exerted while undertaking a specific task.”^99^ While this definition is unclear and subjective, it avoids using ambiguous terminology related to “stress” (which should not be used interchangeably). Although psychological stress can play a role in the overall CWL, evidently workload may increase in the absence of stress.^100^ In light of this, CWL can be defined as the amount of cerebral resources, characterized by neuronal activation and energy utilization, consumed in relation to engagement in a cognitive activity. Adopting this definition would set the foundation for quantifying CWL objectively, allowing the identification of overload or underload states, which might have detrimental implications on patients’ safety and surgeons’ performance.^4,7–10^

Our analysis demonstrated the higher sensitivity of neurophysiologic modalities in detecting CWL. This is likely due to the intrinsic qualities of EEG and fNIRS, such as the capacity for direct measurement of neuronal activation and reduced susceptibility to external influences compared with autonomic measures.^53^ The temporal resolution and sampling rate directly measuring cerebral activity, permits real-time cognitive demand detection, and the use of artificial intelligence (AI) could further facilitate this process.^60,75,101^ Furthermore, while multimodal data analysis is more intricate, evidence from this review and other studies^101^ suggests it enhances the sensitivity for detection of changes in operator workload. Walia et al^75^ integrated EEG and fNIRS to synergistically capture both high temporal-resolution neuronal activity and cortical correlates of microstates, thereby providing a comprehensive assessment of CWL dynamics. This multimodal approach enhances the robustness and depth of insights into cognitive processes during various tasks, and contributes to a more nuanced understanding of workload-related neural phenomena. It is noteworthy that while EEG sensitivity was 100%, the heterogeneity of the reported metrics^59,77,80^ and the lack of unifying frequency band,^52,88,90^ means this should be interpreted with caution.

Of the 7 Eggemeier criteria,^29^ we argue that specificity (selectivity) is most salient: is the sensor actually measuring changes in surgeons’ workload? Physiological objective measures are closely interlinked with autonomic response and thereby heavily confounded by external and internal factors other than CWL (eg, ambient light levels, drugs, and emotional states such as stress, anxiety, and tiredness).^100,102^ In this review, we demonstrated the high sensitivity for HRV metrics, such as SDNN for workload assessment; however, a recent review^21^ concluded that these metrics also correlate with anxiety and perceived stress differences based on STAI and PSS scores (rather than task-related CWL). Indeed, our analysis revealed that the specificity for cardiac and ocular systems was poor. In our view, neurophysiological systems offer a more direct and nuanced understanding of the workload imposed by a task compared with physiological measures linked to autonomic responses, which can be confounded by internal (eg, stress) and external (eg, ambient light levels for pupillometrics) factors.^43,48,91^

Diagnosticity, that is, distinguishing between sources of workload, is invaluable in understanding the specific cognitive demands imposed by different tasks, which would allow targeted optimizations to improve safety by mitigating cognitive challenges in specific domains. An excellent cross-section of task paradigms and CLT domains were identified. Both intrinsic^33,43,46,52,54,55,61,65,67,70,72,79,82–84,88–92,95,96^ and germane load studies^32,35,36,38–42,47,48,50,51,57,58,60,66,68,71,74,75,77,78,80,85,87,93,97^ were prolific and investigated using a range of sensors. As illustrated in Figure 4, a substantial number of studies^44,52,64,73,76^ simultaneously investigated the effects of multiple CLT domains. Despite this, only 5 studies analyzed the effects of individual domains, giving limited diagnostic clarity. fNIRS, HR, and EEG all successfully distinguished sourceload.^52,73,76^

While the literature and modalities’ evolution indicate operator acceptance, the absence of objective data in clinical settings underscores the need for such data to guide operators in choosing the most effective modality. Assessment of intrusiveness—an essential consideration in real-life clinical studies—suggests that peripheral autonomic sensors may be less intrusive than neurophysiology sensors, although this review lacks objective data to support this inference. The practical constraints of implementation involve numerous considerations, including instrumentation, software, and training. Despite technological developments, real-time data analysis is limited by clinical applications (eg, ambient light, environmental distractions, and calibration) and the extensive processing requirements to enable interpretation. AI may play a pivotal role in overcoming these limitations.^60,101^ Advanced AI algorithms can rapidly process complex data streams, and machine learning models can adapt to individual variations optimizing the accuracy of CWL assessments.^60,101^

The studies identified in this review illustrate how CWL can be measured objectively, both in the simulated, and live operating room environments. The applications of these findings in surgery are numerous, including skill acquisition during surgical training, reducing surgical errors, and improving patient safety. By using objective workload data, surgeons can focus their training on specific areas where cognitive demand is high, leading to more targeted and effective training.^84,100,103,104^ Feedback on CWL in simulated environments can give novice surgeons insights into their workload in comparison to experts identifying opportunities to improve skill acquisition as well as developing a neural benchmark of competence.^80^ Furthermore, assessing and understanding the surgical team’s CWL can improve workflow and communication in the operating room, contributing to a more cohesive operating environment and increasing the efficiency of surgical procedures.^49,56^ Finally, the ambitious goal of acquiring real-time feedback on cognitive workload in live operating room environment may have substantial impact on patient safety. Feedback on overload may enable surgeons to make timely adjustments during surgery to maintain optimal performance and reduce the risk of errors.^43^ In addition, it may be used to assist senior trainers in gauging the level of cognitive burden of newly graduated operators in their transition to becoming independent operators.

This review advances the literature on the objective assessment of CWL in surgery by comprehensively analyzing diverse modalities—both autonomic and neurophysiological. Unlike prior reviews of this field,^21,23–26^ the unique contribution of this review is the rigorous examination of psychometric and methodological aspects in the included studies, providing quantitative nuanced insights into sensitivity, selectivity, diagnosticity, and reliability. Furthermore, by addressing the ambiguity in the definition of CW, the review proposes a refined definition grounded in cerebral energy and resource consumption during tasks. The absence of a validated and standardized objective measure for CWL has been thus far ameliorated by the concurrent use of subjective tools such as SURG-TLX. While the goal is to move progressively away from subjective measures, using SURG-TLX enables cross-validation of objective metrics and exploration of relationships between perceived workload and objective data. Finally, adopting a framework for designing studies that assess CWL objectively (including collecting, processing, analyzing, and reporting data) is vital to reach real-time quantification of CWL.

## CONCLUSIONS

Presently, there is no agreed definition of CWL and no validated objective measure of CWL. Our quantitative analysis demonstrates the superior sensitivity of neurophysiological modalities, particularly EEG and fNIRS, in detecting CWL attributed to their direct measurement of neuronal activation and reduced susceptibility to external influences.

A fundamental necessity within this developing field is the establishment of a framework that optimizes study design, allowing for robust comparisons by addressing the heterogeneity in methodological reporting, data processing, and analysis across modalities.

## Supplementary Material
