# Supplementary material for: Objective Assessment of Cognitive Workload in Surgery: A Systematic Review
Source: Ann Surg. 2024 Jun 7;281(6):942–51. doi: 10.1097/SLA.0000000000006370 (PMC12061381; doi:10.1097/SLA.0000000000006370)
Supplement: Supplementary file 1 [file sla-281-942-s001.docx]

**Supplemental Digital Content**

**Supplemental Table 1: key definitions and explanations of the main CWL objective assessment modalities and Eggemier criteria:**

| Glossary | Definitions or description |
| --- | --- |
| Cognitive Workload (CWL) | “The cognitive and psychological effort that is required from a subject to complete a given task” ^1^. |
| Cognitive Load Theory (CLT) | Sweller’s theory posits that an operator has a limited cognitive capacity and that tasks are performed through resource allocation and management ^2^. |
| Source of workload | Description |
| Intrinsic Load | The inherent complexity of the task in question |
| Extraneous Load | External factors and environmental conditions, such as the influence of time pressure, that compete for cognitive resources |
| Germane Load | The cognitive processes related to skill acquisition, pattern recognition, and the development of expertise via schema construction and long-term retention |
| CWL sensor | Description |
| Cardiac metrics | Assess CWL by monitoring physiological changes in the heart's activity. For instance, increased heart rate and reduced heart rate variability are indicative of heightened sympathetic nervous system activity associated with higher CWL states. |
| Heart Rate Variability (HRV) | The variation in time intervals between consecutive heartbeats. |
| HRV - Frequency domain | Assessment of the distribution of heart rate fluctuations at different frequency bands. Higher CWL is often associated with changes in the power spectrum, an increase in low-frequency (LF) and a decrease in high-frequency (HF) components of HRV. |
| Root Mean Square of Successive Differences (RMSSD) | The amount of variability in consecutive heartbeats that is related to the high-frequency components of HRV |
| HRV - Time domain | Assesses CWL by analysing the variations in time intervals between successive heartbeats. Increased CWL often correlates with reduced variability in these beat-to-beat intervals, reflecting greater sympathetic nervous system activity. |
| HRV - non-linear parameters | Assessment of complex patterns/irregularities in the heart rate fluctuations. Changes in non-linear dynamics, such as fractal-like behaviour or entropy, offer additional insights into the complexity and adaptability of the autonomic nervous system. |
| SampEn and ApEn | Sample Entropy and Approximate Entropy used in the non-linear analysis of time-series data, particularly in assessing the complexity or irregularity of HRV. |
| Ocular metrics | Assess CWL by tracking eye movements, pupillary changes, gaze patterns and fixations provide valuable insights into how individuals allocate attention and process information, offering a quantitative measure of cognitive workload during tasks. |
| Gaze | Assesses CWL by examining how surgeons allocate visual attention and process information during procedures. Increased gaze distance or entropy, along with variations in gaze velocity, can signify heightened cognitive demands. |
| Fixations | Assesses CWL by measuring the duration and locations where a surgeon's eyes come to a temporary halt during a procedure. Prolonged or frequent fixations may indicate increased cognitive demands as surgeons allocate focused attention to specific areas. |
| Pupillometry | Assesses CWL by analysing changes in pupil size, which is indicative of variations in mental effort and arousal. Larger pupil dilation often corresponds to heightened CWL, providing a non-intrusive and real-time measure of the surgeon's cognitive engagement and focus during different phases of a surgical procedure. |
| Saccade | Assesses CWL by evaluating the rapid eye movements between fixations, reflecting the efficiency of visual scanning and information processing. Increased CWL in surgery may manifest through alterations in saccadic patterns. |
| Blink patterns and Percentage of Eye Closure (PERCLOS) | Increased CWL can lead to changes in blink frequency and PERCLOS, with decreased blink rates and prolonged eyelid closure. |
| Ocular Area of Interest | Assessment of where a surgeon directs their visual attention and duration of focus on specific areas. Prolonged dwell times or irregular scan paths indicate increased CWL. |
| Functional Near Infra-Red Spectroscopy (fNIRS) | Assesses CWL by detecting changes in cerebral oxygenation and hemodynamics, reflecting the metabolic demands of increased neural activity during cognitive tasks. |
| Delta HbO2 | Assesses CWL through the precise measurement of changes in the concentration of oxygenated haemoglobin (HbO2) within the prefrontal cortex. Dynamic alterations in [Delta HbO2] are indicative of the metabolic response to increased neural activity. |
| Delta HHb | Assesses CWL by detecting changes in the concentration of deoxygenated haemoglobin (HHb) in the prefrontal cortex. Reduction in Delta HHb signify a change in cellular oxygen extraction and consumption, reflecting the metabolic response to neural activity. |
| Electro Encephalography (EEG) | Assesses CWL by directly detecting electrical brain activity, specifically changes in frequency and amplitude, providing insights into the neurophysiological correlates of cognitive engagement. For instance, increased theta and alpha power, along with changes in event-related potentials, can serve as markers of CWL. |
| EEG Alpha | (8-12Hz) has also been associated with CWL, cognitive fatigue and inattention |
| EEG Beta | (12-30Hz) is considered more specific for working memory, visual attention and short-term memory. |
| EEG Theta | (4-8Hz) has been associated with workload, increased working memory and tasks associated with sustained concentration. |
| Hormonal | Reflects the physiological stress response and adaptive hormonal changes associated with increased mental demands. Elevated cortisol levels may indicate heightened stress and CWL, while changes in testosterone levels may provide insights into the neuroendocrine response, collectively characterising the surgeon's physiological state. |
| Pulmonary | Assesses CWL by reflecting changes in autonomic arousal and physiological stress response. Increased respiratory rate or altered breathing patterns may indicate heightened cognitive demands and stress levels. |
| Eggemier Criterion | Description |
| Sensitivity | “Capacity of the instrument to detect changes in task difficulty or cognitive demands” |
| Diagnosticity | “Capacity of the instrument to differentiate distinct sources of workload, such as specific capacities or multiple resources” |
| Selectivity/Valididty | “Sensitivity of the instrument only to differentiate in cognitive demands, not changes in other variables(e.g., physical workload, stress” |
| Reliability | “Consistent assessment of mental workload” |
| Intrusiveness | “Lack of interference with primary task performance” |
| Implementation requirements | “Practical constraints associated with instrumentation, software, and training” |
| Operator acceptance | “Operator perception of the validity and usefulness of the procedure” |

*Subjective measures are retrospectively reported by the user and include: NASA Task Load Index (NASA-TLX) and the surgery task load index (SURG-TLX).*

*Objective measures are collected in realtime, and include:*

1. *Physiological changes:*
   1. *cardiac electrical activity measured with ECG.*
   2. *Ocular parameters including pupil size, gaze behaviours and blink metrics.*
2. *Neurophysiological changes:*
   1. *neuronal electrical activity measured with EEG*
   2. *haemodynamic changes measured with fNIRS*

**Supplemental Table 2: Summary of search terms used**

| **MeSH terms** | **Non-MeSH terms** | | |
| --- | --- | --- | --- |
|  | **CWL** | **Assessment** | **Surgery** |
| Stress, Psychological  Physiological stress Workload  Cognition  Cognition assessment  Cognitive Reserve  Surgeons  Anesthetists  Radiologists  Interventional radiologist  Operating Rooms  Wearable Electronic Devices  Electrocardiography  Electromyography  Electroencephalography | Stress  workload  cognitive* | Wearable*  Sensor*  GSR  Galvanic skin response  Skin conduct*  ECG  Electrocardiogra*  EMG  Electromyogra*  EEG  Electroencephalogra*  Heart rate  Pupil  Gaze  eye track*  objective assessment*  SURG-TLX  NASA-TLX  SWAT  NIRS  FNIRS  functional near infrared spectroscopy Magnetoencephalogr*  MEG  Accelerometer  Head positioning  Saccade | Surgeon*  Intraoperative  Anesthetist*  Anesthesiologist*  Radiologist*  Operation theatre* Operation room*  Operating room*  Operating theatre* |

*MeSH, Medical Subject Heading;*

*Truncation symbols (*),* *used as a substitute for any string of zero or more characters at the end of a word;*

*Wildcard symbol (?) can be used as a substitute for one character or none;*

*Adj2, adjacency searching for both terms and up to one word in between them*

*We used "AND" conjunction between the different three categories using MeSH and Non-MeSH terms: "CWL" terms AND "Assessment" terms AND "Surgery" terms*

**Supplemental Table 3: Inclusion and exclusion criteria for the search**

| **Inclusion** | **Exclusion** |
| --- | --- |
| Population: surgeons, anaesthetists or interventional radiologists | Population: non-surgical |
| Study setting:   1. Reported the use of an objective measure for CWL detection 2. The task paradigm ensures mental demand is altered (i.e., either increased or reduced) or comparing two task paradigms with different mental demands | Setting:   1. Does not report an objective measure 2. The task paradigm does not result in change in CWL or change in CWL cannot be assessed |
| Study designs: any | Commentaries, Editorials, Letters, Abstract only published, Conference posters and Case report |
| Study language: English only | Language other than English |
| Study period: any | N/A |

**Supplemental Table 4: Generic data extraction template for all studies**

| Study Author, year | N | Specialty | Population | Simulation or real life | Task Paradigm | Stressor or de stressor task type | CLT domain | Device | Region | Objective measurement | Correlation with task load | Subjective measurement | Correlation with task load | Correlation of subjective with objective metric(s) | CWL definition | Summary |
| --- | --- | --- | --- | --- | --- | --- | --- | --- | --- | --- | --- | --- | --- | --- | --- | --- |

**Supplemental Table 5: Newcastle-Ottawa quality assessment**

| Study (First Author, year) | Selection of study group | | | | Comparability of study groups | Outcome | | | Overall score |
| --- | --- | --- | --- | --- | --- | --- | --- | --- | --- |
|  | Representativeness of exposed cohort | Selection of non-exposed cohort | Ascertainment of exposure | Demonstration that the outcome of interest was not present at the start of the study | (**) | Assessment of outcome | Follow-up long enough for outcomes to occur for >30 days | Adequacy of follow-up |  |
| Kennedy-metz 2022 | * |  | * | * | ** | * |  | * | Good |
| Kelkar 2022 | * | * | * | * | * | * |  | * | Good |
| Yu 2022 | * | * | * | * | * | * |  | * | Good |
| Abdelrhaman 2016 | * | * | * | * | ** | * |  | * | Good |
| Bakhsh 2018 | * | * | * | * | ** | * |  | * | Good |
| Dedmon 2019 |  |  | * | * | * |  |  | * | Poor |
| Dias 2023 | * | * | * |  | ** | * |  | * | Good |
| Heemskerk 2014 | * | * | * |  | ** |  |  | * | Fair |
| Martin 2016 | * | * | * | * | * | * |  | * | Good |
| Pimentel 2019 | * | * | * | * | * | * |  | * | Good |
| Rieger 2014 | * | * | * | * | * | * |  | * | Good |
| Weinger 2004 | * | * | * | * | * | * |  | * | Good |
| Zakeri 2020 | * | * | * | * | * | * |  | * | Good |
| Carnevali | * | * | * | * | ** | * |  | * | Good |
| Hinzmann 2023 |  | * | * |  | ** | * |  | * | Fair |
| Mazzella 2023 |  | * |  | * | * | * |  | * | Fair |
| Paljewala 2023 |  | * | * | * | * |  |  | * | Fair |
|  |  |  |  |  |  |  |  |  |  |
|  |  |  |  |  |  |  |  |  |  |
|  |  |  |  |  |  |  |  |  |  |
|  |  |  |  |  |  |  |  |  |  |
| Izzetoglu 2021 | * | * | * | * | * | * |  | * | Good |
| Modi 2019 | * | * | * | * | * | * |  | * | Good |
| Modi 2020 | * | * | * | * | ** |  |  | * | Good |
| Modi 2018 | * | * | * | * | * | * |  | * | Good |
| Nemani 2018 | * | * | * | * | * | * |  | * | Good |
| Singh 2018 | * | * | * | * | * | * |  | * | Good |
| Leff 2008 | * | * | * | * | * | * |  | * | Good |
| Crewther 2016 | * | * | * | * | ** | * |  | * | Good |
|  |  |  |  |  |  |  |  |  |  |
| Guru 2015 | * | * | * | * | * | * |  | * | good |
| Guru 2015 [2] |  |  | * | * | ** | * |  | * | fair |
| Hussein 2016 |  |  | * | * | * | * |  | * | fair |
| Maddox 2015 | * | * | * |  | * | * |  | * | good |
| Maimon 2022 | * | * | * |  | * | * |  | * | good |
| Morales 2019 | * | * | * |  | * | * |  | * | good |
| Plazak 2019 |  |  | * |  | * | * |  | * | poor |
| Shafiei 2016 |  |  | * | * | ** | * |  | * | fair |
| Shugaba 2023 | * | * | * | * | * | * |  | * | Good |
| Suarez Revelo 2019 |  | * | * |  | * | * |  | * | fair |
| Yang |  |  |  |  |  |  |  |  | Good |
| Yu 2022 | * |  | * | * | - | * |  | * | poor |
| Walia 2022 |  |  |  |  |  |  |  |  | Good |
| Wu 2021 | * | * | * | * | ** | * |  | * | good |
|  |  |  |  |  |  |  |  |  |  |
| Anschueutz 2019 | * | * | * | * | ** | * |  | * | Good |
| Berges 2022 | * | * | * | * | * | * |  | * | Good |
| Cai 2022 | * | * | * | * | * | * |  | * | GOod |
| Berguer 2001 | * |  | * | * | * | * |  | * | Good |
| Diaz-Piedra 2017 | * |  | * | * | * | * |  | * | Good |
| Distasi 2017 | * | * | * | * | ** | * |  | * | Good |
| Erridge 2018 | * | * | * | * | ** | * |  | * | Good |
| Gao 2018 | * | * | * | * | ** | * |  | * | Good |
| Nguyen 2019 | * | * | * | * | ** | * |  | * | Good |
| Richstone 2010 | * |  | * | * | * | * |  | * | Good |
| Schulz 2011 | * | * | * | * | * | * |  | * | Good |
| Schulz 2014 | * | * | * | * | * | * |  | * | Good |
| Wu 2020 | * |  | * | * | * | * |  | * | Good |
| Zhang 2017 | * |  | * | * | * | * |  | * | Good |
| Zhang 2018 | * | * | * | * | * | * |  | * | Good |
| Zhang 2021 | * | * | * | * | * | * |  | * | Good |
| Zheng 2012 | * | * | * | * | * | * |  | * | Good |
| Zheng 2015 | * | * | * | * | * | * |  | * | Good |
| Bednarik 2018 (Blink) | * | * | * | * | * | * |  | * | Good |
| Bednarik 2018 (Pupil) | * | * | * | * | * | * |  | * | Good |
| Cao 2016 | * | * | * | * | ** | * |  | * | Good |
| G. Dalveren 2017 | * |  | * | * | * | * |  | * | Good |
| G. Dalveren 2018 | * |  | * | * | * | * |  | * | Good |
| Dalveren 2018 (ECE) | * | * | * | * | * | * |  | * | Good |
| Di Stasi 2017 | * | * | * | * | ** | * |  | * | Good |
| Dilley 2020 | * | * | * | * | ** | * |  | * | Good |
| Gao 2019 | * | * | * | * | ** | * |  | * | Good |
| Gunawardena 2019 | * | * | * | * | ** | * |  | * | Good |
| Gunawardena 2019 | * | * | * | * | ** | * |  | * | Good |
| Jiang 2013 | * |  | * | * | * | * |  | * | Good |
| Jiang 2014 |  |  | * | * |  | * |  | * | Fair |
| Koskinen 2020 | * | * | * | * | ** | * |  | * | Good |
| Nguyen 2020 | * | * | * | * | ** | * |  | * | Good |
| Ortega Moran 2019 | * | * | * | * | ** | * |  | * | Good |
| Tien 2015 | * | * | * | * | ** | * |  | * | Good |
| Wu 2021 | * |  | * | * | * | * |  | * | Good |
| Liu 2023 | * |  | * | * |  | * |  |  | Poor |

**Supplemental Table 6: Summary of cardiac studies**

| CLT domain | Author year | Setting | Task paradigm | Parameter | Results | Study summary and notes |
| --- | --- | --- | --- | --- | --- | --- |
| Extraneous load | Kennedy-Mets 2022 | Real-life | Continous noise level from the operating theatre and continous heart rate data were taken from the core surgical team during on-pump cardiac surgery (14 CABG, 4 AVR) | TIme domain: Normalised Team HR during 18 high-noise 5-minute segments compared 18 low-noise 5-minute segements. | High noise level was significantly associated with high team HR (p<0.001), and a higher percentage of time reflecting case-irrelevant communication events was associated with higher-noise segments compared to lower-noise segments (t(34) = -2.105, p = 0.021) | Ambient noise (extrenous load) during the seperation from bypass phase of cardiac surgery is considered the stressor. The study showed that HR (normalised team HR) correlates positively with noise, especially case-irrelevant communication. |
|  | Palejwala 2023 | Real life | Participants performed burn surgery (No difference in duration and TBSA of surgeries) under thermoneutral condition and hot conditions. | Time domain: Mean HR | Extrenous load from increased ambient temperature was significantly associated with higher mean HR (103 BPM compared to 95 BPM, P<0.01) | 10 participants performed burn surgeries in HOT temperature (30.8±1.1 C) and CON temperature (24±1.1 C), representing extrenous cognitive load. There was significant difference in core body temperature SURG-TLX showed significant difference (P<0.01) which was consistent with the difference in Mean HR (P<0.001). |
| Intrinsic load | Abdelrahman 2016 | Real life | Maximum HR of one surgeon was taken during 23 SILC and 25 CLC | Time domain: Max HR | Post opertaive maximum HR was 13.74% higher (p=0.02) in SILC than CLC and change in maximum HR between post operative and intraoperative timepoints was more than 100% higher in SILC (p=0.02) | SILC requires higher cognitive workload as measured by Surg-TLX. Post operative max HR was significantly higher in SILC group. Intra-operative cortisol levels were significantly higher in SILC group. |
|  | Heemskerk 2014 | Real life | Objective measures were taken from the particiapnts during the procedures (laparascopic cholecyctsectomy and robotic cholecystectomy) | "Time domain: Mean HR Frequency domain: HRV (LF/HF ratio)" | Baseline is equal for both groups, but in the course of the operation, CC leads to a significant higher mean heart rate compared to the base- line level, whereas RC leads to a lower HR compared to the baseline level. During the operation, CC leads to a significant higher LF/HF ratio than RC. Interestingly, the LF/HF ratios for RC are significantly lower than those for CC during stages 4, 5 and 6 of the operation, even despite the rather small numbers of op- erations as can be seen in figure | The study Hypothesised that robotic assistance leads to a HRV comparable to open surgery in surgeons with comparable expertise. The results show that the mean heart rate during RC is lower compared to CC. This suggests that the use of robotic assistance for this procedure reduces total strain, which is the combined physical and mental strain. LF/HF ratio was lower when the surgeon used robot-assistance for the cholecystectomy, suggesting that laparoscopic procedure leads to significantly higher mental strain compared to robotic. |
|  | Dedmond 2019 | Real life | Objective measures were taken from the particiapnts during the procedures (Mastoidectomy < Facial Nerve dissection) on cadavers and on real patients. | "Time domain: Mean SDNN Frequency domain: HRV (LF/HF ratio)" | No statistically significant differences in mean heart rate were observed among the preoperative, mastoidectomy, and facial nerve dissection periods. Mean SDNN values during resting were 43.9 􏰆 9.2 ms and decreased to 34.1 􏰆 8.9 ms preoperatively ( p 1⁄4 0.13) (Fig. 3). A continued decline in SDNN was observed in the operating room during mastoidectomy to 29.4 􏰆 11.7 ms ( p 1⁄4 0.06 compared to resting) and 22.8 􏰆 3.1 ms during facial nerve dissection ( p 1⁄4 0.002 compared to resting, p 1⁄4 0.03 compared to preoperative). Facial nerve LF/HF was significantly increased compared to resting values (p = 0.004) but the increase was not significant com- pared to preoperative values ( p = 0.48). | Task complexity was hypothesised to be the stressor. Mean HR showe no statistical difference. Mean SDNN was significantly lower between FN dissection and resting, but not other stages. Mean LF/HF ration during FN dissection was significantly higher than resting, but not other stages. |
|  | Kelkar 2022 | Real-life | Mean HR and Mean SPO2 were taken from three surgeons during phacoemulsification cataract surgery using a three-dimensional system compared to using conventional microscope surgery. | "Time domain: Mean HR Saturation: Mean SPO2" | Surg TLX showed no significant difference in CWL between . Heart rate (Mean HR) and SPO2 (Mean SPO2) showed no significant difference. | Surg TLX showed no significant difference in CWL between 3D and conventional Microsscopic Phaco-emulsification. Heart rate (Mean HR) and SPO2 (Mean SPO2) also showed no difference. |
|  | Martin 2016 | Real life | Objective measures were taken from the participants during induction, maintenance and emergence. | "Time domain: Mean HR, SDNN, RMSSD, pNN50 Frequency domain: LF/HF ratio, LF power [%], LF power, HF power Non-linear: PeEn, SampEn, ShanEn, D2 " | CWL differed significantly between stages of anaesthesia (Induction and emergence vs maintenance): | This study measures linear and non-linear (exploratory analysis) of mental workload. They established difference in CWL between stages of anaesthesia by Borg and NASA-TLX. HR and the HRV metrics of the time domain (all) differed significantly between the different anaesthesia stages. In the frequency domain, only the relative and the absolute LF power and the absolute HF power differed significantly between the stages. The parameters PeEn, SampEn, ShanEn and D2 that are based on the non-linear HRV analysis, differed significantly. Logistic regression shows higher AUC in models based on non-linear heart rate metrics compared with linear metrics. |
|  | Pimentel 2019 | Real life | Objective measures were taken from the participants during aneurysm clipping procedure. This review focused on aneurysm rupture | "Time domain: AVNN, SDNN, pNN20, pNN50. Frequency domain: LF/HF ratio. Non-Linear: 1V, 2LV, SD1, SD2, CVI, CSI" | Aneurysm rupture resulted in low variability (pNN20=0.0%, pNN50=0.0%) | This study had 2 neurosurgeons, although only one analysed performing aneurysm clipping. The cognitively demanding stages were determined through Surg-TLX, with aneurysm rupture having high CWL. The relationship between HRV (Both linear and non-linear) was then calculated. Interstingly LF/HF ratio did not correlate with SURG-TLX. |
|  | Schulz 2011 | Simulation | Objective measure were taken form anaesthestic doctors in clinical scenarios (x3) during induction. Scenario 1: familiarisation; no critical incident. Scenario 2 or 3: In a randomized order, workload was increased by simulation of a critical incident | Time domain: Mean HR | In uneventful sessions, the weighted mean heart rate was 95 beats min21, and in incident sessions, it increased to 103 min21 [P1⁄40.115; 95% CI of difference (23, 20)]. Overall, the fit by the mixed model is highly significant (P,0.001). | Mean heart rate of 15 anaesthetic trainees recorded during three simulation scenarios with varying levels of workload. HR reflected workload within simulator sessions. |
|  | Song 2009 | Real life | Objective measures were taken from the attending while they performed coronary artery bypass grafting (CABG) surgery and when he supervised and assisted CABG | Frequency domain: LF/HF ratio | When performing the operation, The ratio of low frequency to high frequency was at a peak in the beginning of the operation and gradually decreased toward the end of the operation. In contrast, when he assisted a resident, the ratio of low frequency to high frequency was at a peak in the phase of aortic cross-clamp, coronary anastomosis, and unclamping. | The study assumes HRV is an indirect measurement of CWL. When an attending-consultant surgeon performed the operation himself, the most anxious part of the operation was at the beginning and thereafter the level of anxiety gradually declined. In contrast, when he assisted a resident, the highest level of anxiety was when the aortic cross-clamp was in place and out of place and during the coronary anastomosis. The task does not have a specific stressor and the results are explained retrospectively. |
|  | Weigner 2004 | Real-life | Objective measures were taken from the participants during induction, maintenance and emergence in teaching and non-teaching settings. | Time domain: Mean HR, minimum HR and maximum HR. | Teaching vs non-teaching: No significant difference. Phase of anaesthesia: higher in induction and emergence compared to maintenance (p<0.05) | CWL (As per Borg scale and response-latency) was higher during induction and emergence in teaching cases, compared to non-teaching. This was not reflected in average, maximum or minimum HR.  CWL was higher in induction and emergence compared to maintenance. Mean and maximum (but not minimum) HR was increased during induction compared with maintenance phase (P< 0.05). The increase was not significant in emergence. |
|  |  |  | Compared Junior residents with experienced clinicians | Minute by minute HR | Higher in junior residents compares to intermediate and experienced. | Intermediate residents had a significantly lower HR peak at the time of patient intubation compared with junior residents (P<0.05). Experienced clinicians had the lowest HR values throughout the anaesthetic that were significantly less (P<0.05) than the HR of inexperienced providers at many time points during both the induction and emergence. |
| Germane Load | Hinzmann 2023 | Simulation | Objective measures were taken while participants performed simulated cerebral angiography | "Time domain: Mean HR, SDNN, RMSSD, NN50, pNN50 Frequency domain: VLF%, LF power [%], HF power [%], LF [nu], HF [nu], VLF abs, HF, LF/HF " | From the time-domain HRV, RMSSD, p = 0.039, NN50, p=0.007, and pNN50, p=0.012, differed significantly between experts and novices. From the frequency domain, VLF%, p=0.006, HF%, p=<0.001, LF/HF ratio, p=<0.001, LF nu, p=0.001, HF nu, p= 0.001, HF abs, p=0.019 differed significantly between experts and novices. | 11 participants (6 novices and 5 experts) perfored simulated diagnositic angiography. HRV and NASA-TLX used to assess the effect of expertise (germane load). NASA-TLX mental demand scores did not differ between groups (P=0.338). HRV results were inconcordant. From the time-domain HRV, RMSSD, NN50, and pNN50, differed significantly, and from the frequency domain, VLF%, HF%, LF/HF ratio, LF nu, HF nu, and HF abs differed significantly. When using a linear regression model, pNN50 was the only metric connected with expert status from the time-domain, while all 5 metrics of frequency domain were connecetd. |
|  | Bakhsh 2019 | Simulation | Objective measures were taken from the participants during the simulation (TEVAR in 4 stages - baseline, verbal rehearsal, individual simulation, Team simulation) | "Time domain: Mean HR Frequency domain: LF/HF ratio" | Junior surgeons experienced significantly higher mean HR and signifanctly higher HRV compared to senior surgeons (82 ± 5.83 vs 76 ± 6.02, p=0.033 and 8.32 ± 2.84 vs 5.57 ±1.90, p<0.001). | NASA-TLX and HR (HRV, Mean HR) were measured for junior and senior surgeons during individual and team simulation. Senior surgeons experienced lower HR and HRV (LF/HF ratio) compared to Junior surgeons. HRV (LF/HF) correlated significantly with CWL measured by NASA-TLX. |
|  | "Carnevali 2023 " | Real life | Participants performed real life surgeries (various, uncontrolled) | Time domain: Mean HR and RMSSD | HRV (RMSSD) reduced significantly from baseline during the surgery; howerever there was no significant difference between seniors and experts | HRV (RMSSD) and cortisol measures between experts and seniors were compared during surgical real life surgical procedures. Althogh there was a significnat increas in mean HR and decrease in RMSSD, there was no significant difference between the two cohorts. |
|  | Schulz 2014 | Simulation | Objective measure were taken form anaesthestic doctors of different expertise in clinical scenarios (x3) during induction. Scenario 1: familiarisation; no critical incident. Scenario 2 or 3: In a randomized order, workload was increased by simulation of a critical incident. | Time domain: Mean HR | Incraese in Mean HR in the inexperienced group was insignificant (P=0.367) | Retrospective analysis of Schulz 2011. Hypothesised experienced anaesthetists increase in workload is less than less experienced. T tests and multivariate generalized linear models (MANOVA) used for retrospective analysis. Performance did not depend on experience. Mean HR increase was not significant. |
|  | Yu 2022 | Simulation | "Students conducted four pre and post experiments in the training box. They were trained on VR-based laparoscopic surgery simulators (VRLS) in the middle of pre and post experiments. " | Time domain: Mean HR, Maximum HR. | "The average heart rate decreased sig- nificantly (p < 0.05) The maximum heart rate dropped at the post-test procedure (p < 0.05)." | Training session was used to optimise germane load in the experimental group. NASA-TLX and WP scale showed that CWL decreased following training with significant improvement in performance score. Subjective measures show a significant reduction in CWL post FT and post CRT. Correlatiobn between subjective and objective measures show positive correlation between CLS (cognitive load score computed from EEG) and CLE (Cognitive load computed from EEG). Pearson correlation between surgical skills and heart rate was did not show a significant correlation. |
|  | Reiger 2014 | Real-life | Objective measures were taken from the participants during an ordinary operating day. | Time domain: Mean HR | No difference in mean HR for main surgeon vs assistant (p = 0.668; η2 = 0.011) | This study used physiologic parameter (HR, BF) and subjective (NASA-TLX) to assess CWL. They concluded: Subjectively: primary physicians are more stressed before and after procedure. Lenghth of surgery had a significant impact on on temporal and mental demands. Objective measurements showed no difference in Mean HR and BF in surgical roles. Length of surgeries tended to have an impact on intraoperative mean HR but not on BR. |
| Uncontrolled | Mazzella 2023 | Real life | Two surgeons performed 10 Robot-assisted thoracic surgery procedures (RATS) and 10 open thoracic surgery procedures - uncontrolled | Time domain: Mean HR, Maximum HR, Minimum HR Respiratory Rate: Mean RR, Maximum RR, Minimum RR | "Mean, maximum and minmum HR were significantly lower in RATS (P<0.001). Mean, maximum and minimum Respiratory rate were also significantly lower (P=0.42, P=0.22, and P=0.65 respectively) " | There was lower cognitive workload with RATS compared to open as measured by NASA-TLX. Objective HR metrics (Mean, Maximum and Minumum) were lower in RATS reflecting lower CWL. Similarly, Mean, Maximim and Minimum Respiratory rate were lower in RATS group. |
|  | Dias 2023 | Real life | 4 perfusionists perfused patients during the CardioPulomary Bypass (CPB) phase of cardiac surgery | Frequency domain: HF power [%], HF [nu], LF [nu], LF power [%], LF/HF ratio, VLF peak frequency, DFA alpha1 | HRV metrics HF relative power and HF absolute power correlated with SURG-TLX (r=-0.491, P=0.003 and r=0.485, P=004) resepctively | The task paradigm was uncontrolled and no change in reported/expected. CWL was measured using SURG-TLX. The results show that only the HF relative power and HF absolute correlate with the pereceived workload as measured by SURG-TLX. |
|  | Zakeri 2020 | Simulation | Novice and experienced participants were asked to perform a task. As they performed tasks on a LS trainer box, the subjects responded to randomly timed auditory stimuli by pressing a pedal. | "Time domain: Mean HR Frequency domain: HF / (HF+LF)"  Blink Rate | Unable to extract - Pooled data | Partcipants performed simulated controlled tasks. However, data was pooled, therefore deemed uncontrolled. Cardiac metrics were extracted from fNIR signals and Eye metrics from EEG signals. NASA-TLX was used as baseline. Pearson correlations show that HR’s mean and standard deviation tended to increase with the experienced difficulty as measured by the NASA-TLX score. HRV appeared to be an even better predictor. The minimum value of HRV was significantly negatively correlated with the NASA-TLX score. Full set of features collectively outperformed any individual feature in predicting subject's experience and perfomrance. |
| Uncontrolled * |  |  |  |  |  |  |

**Supplemental Table 7: Summary of ocular studies**

| CLT domain (or uncontrolled) | Study Author, year | Simulation or real life | Task Paradigm | Objective measurement | Results | Summary of the study (task paradigm, objective measure and findings) |
| --- | --- | --- | --- | --- | --- | --- |
| Germane | Berges 2023 | Simulation | Pointing to 9 endoscopic targets during Cadeveric Endoscopic Sinus Surgery | "Gaze metrics: distance, velocity AOI: scan path (density, duration, length) Saccades: number, duration Fixations: number, duration, fixation ratio" | All metrics with the exception of fixations’ mean duration and time to the first fixation, showed significant difference between novice and expert participants (p<0.0001). | Eye gaze, endoscope, and tool motion data can provide an objective and accurate measurement of ESS surgical performance. Incorporation of these algorithmic techniques intraoperatively could allow for automated skill assessment for trainees learning endoscopic surgery. Saccade:fixation ratio and total fixation duration were metrics conserved across all tasks used to predict skill. In contrast, fixations’ mean duration and time to the first fixation, were not different between skill levels. |
| Germane load | Bednarik 2018 | Simulation | Simulated microscopic suturing - divided into subsegments (﻿needle pick, edge touch, pierce, needle push, extraction, thread handling, throws/knots 1, 2, 3, and cutting) | Pupillometry: Percentage change in pupil dilation; blink rate | There was a significant effect of expertise level (F(1, 1070) = 35.789, p < .001, η2 = .032); the interaction effect was also significant (F(9, 1070) = 5.714, p < .001, η2 = .046), indicating that pupil dilated at different segments of a suture depending on the expertise: at the beginning experts’ pupil dilated more than novices’, while starting with the knotting the situation changed. | Novice and expert surgeons performed various microscopic suturing tasks. Change in pupil dialtation was significant between experts and novices indicating that larger pupil dialation refelcts larger CWL utilised. |
| Germane load | Cai 2022 | Simulation | Simulated hip arthroscopy: Establish 4 arthroscopy portals, and identify the 12 o'clock, 2 o'clock, and 4 o'clock positions on the labrum | "AOI: Dwell time Fixations: number Saccade: number, duration, peak velocity Pupillometry: pupil entropy" | Dwell time, No. of fixations, Saccade duration, Peak saccade velocity, and Pupil Entropy showed significant correlation with the level of expertise. | A correlation was identified between the eye tracking metrics and participants’ experience levels. Hence, it is feasible to apply eye tracking data as a supplementary objective assessment tool to benchmark the technical proficiency of surgical trainees in hip arthroscopy, and enhance simulation-based training |
| Germane load | Cao 2016 | Simulation | Simulated intracorporal knot-tying x 4 blocks | Pupil dilation |  | The more proficient surgeons exhibited a smaller distribution in pupil diameter than did the less proficient surgeons and that an individual’s suturing proficiency is closely related to the degree of dispersion of pupil diameter. |
| Germane load | Dilley 2020 | Simulation | Continous suturing task on avian tissue model via da Vinci robot | "Fixations: number, average duration, Maximum duration, frequency Saccades: Number, Length, frequency, maximum velocity, average velocity, average durations Pupillometry: maximum size right eye, average size left, average size right eye, average size both eyes, Rate of diameter change for left eye, Rate of diameter change right eye, size entropy left eye, size entropy right eye Eyelid metrics: Number of blinks over total time period, Number of blinks per minute " | Significant (P ≤ .05) correlations were seen between pupil size, rate of change and entropy, and associated GEARS/NPMTS in “novice” and “proficient” surgeons. Only number of blinks per minute was significantly different between pupilometrics in the simulated and real tissue environments | Participants completed continous suturing task on avian tissue model via da Vinci robot as novices. This was followed by training until they all achieved the expert-derived benchmark of proficiency threshold twice consecutively or five times non- consecutively to become "experts". They then performed one further continous suturing task on an avian tissue model. Eye metrics significantly correlate with profeciency and could be incorporated into robotic surgery assessments. |
| Germane load | Erridge 2018 | Real life | Laparoscopic Roux en Y gastric bypass divided into 4 segments: preoperative set-up including port placement and liver retraction (segment 1); dissection of the angle of His (segment 2); formation of the retro- gastric tunnel (segment 3); and construction of the gastric pouch (segment 4). | "AOI: Dwell time Fixation: frequency Eyelid metrics: Blink rate Pupilometry: Max pupil size, Rate of diameter change " | "Compared with juniors, experts showed a longer normalized dwell time on the screen vs juniors in all segements (P=0.004, P=0.001, P=0.001, P<0.001), smaller fixation frequency in all segments (P=0.023, P=0.036, P=0.011, P=0.011), smaller blink frequency in all segments (P < 0⋅010, P < 0⋅010, P < 0⋅001, P < 0⋅001), maller rate of pupil range in all segments (P < 0⋅010, P < 0⋅010, P < 0⋅001, P < 0⋅010), and smaller maximum pupil diameter in segment 2 ,3, and 4 (P < 0⋅010). " | "Juniors and experts performed laparoscopic RYGB (divided into 4 segments). Experts had a longer normalized dwell time on the screen, smaller fixation frequency, smaller blink frequency, smaller rate of pupil range, and smaller maximum pupil diameter indicating lower CWL. However, In segments 1 and 4, juniors had significantly more fixations per second on the operating theatre and on the nurses in segment 4 indicating different behaviours. " |
| Germane Load | Gao 2018 | Simulation | Laparoscopic appendicectomy in noisy room vs silent vs music. | Pupilometry: change in pupil diameter | The experienced operators had significantly smaller pupil dilation than the moderately experienced operators under silent conditions (P=0.011) and under noise conditions (P=0.004). | "There was no sig difference in NASA-TLX or performance between experienced and moderately experienced participants under all conditions. However, Experienced operators had significantly smaller pupil dilation than the moderately experienced operators under silent conditions (P=0.011) and under noise conditions (P=0.004). " |
| Germane load | Gunawardena 2019 (2) | Real life | Laparoscopic cholecystectomy | "Saccade: Average amplitude Fixation: Number, duration Pupilometry: Change in Pupil Diameter (Percentage) Index of Pupillary Activity (IPA)" | "Experts have a lower number of fixations (ANOVA F1,37 = 21.84, p < 0.001), higher fixation duration (ANOVA F1,37 = 8.883,p < 0.001), and lower IPA (0.083 (SD=0.019)) vs (0.112 (SD=0.018)) vs (0.099(SD=0.016)) in experts, non-experts and semi experts respectively. There was no difference in avergae saccade amplitute (ANOVA F1,37 = 0.445,p = 0.509) and percentage change in pupillary diameter (ANOVA F1,37 = 0.445,p = 0.509). " | "4 participants (novice, semi-expert and expert) performed laparascopic cholecystectomy. The results showed that fixation number, duration, and the index of pupillary activity (IPA) can use to differentiate the skill levels during live surgeries. " |
| Germane load | Koskinen 2020 | Simulation | Microscopic suturing task on a training task board was designed iteratively by experienced surgeons | "Eyelid Metrics: Blink rate Pupillometry: change in pupil diameter" | The mean blink rate per suture was 4.69 blinks/min (SD = 5.04) for experts and 4.68 blinks/min (SD = 3.62) for novices. Percentage change in pupil size, SD of pupil size change, and blink rate allow suture-based classification of expertise with 74.3%-76% accuracy. | 6 novices and 5 microsurgeons performed suturing tasks, percentage change in pupil size was used to classify expertise according to a support vector machine classifier. Classification of expertise from sutures achieved accuracies between 74.3% and 76.0%. Classification from individual suturing segments based on these same features was not feasible. |
| Germane load | Nguyen 2020 | Simulation | Robotic tasks: EndoWrist® manipulation, needle targeting, suturing/knot tying, and excision/dissection. | TEPR Quantified by the Index of Cognitive Activity (ICA) | Decrease in ICA as surgeon experience increased. This trend exhibited signifi- cance across three out of the four dry lab exercise types: EndoWrist® Manipulation (p < 0.005), suturing/knot tying (p < 0.037), and excision/dissection (p < 0.043). | 26 participants (experts, intermediate and novices) performed simulated robotic tasks. Automated performance metrics and task evked pupillary responses were used to classify expertise, they were also compared to each other. Task-evoked pupillary response (ICA) distinguished surgeon expertise for three out of four task types (p<0.04). |
| Germane load | Schulz 2014 | Simulation | Critical events during anaesthesia induction: Scenario 1: familiarisation; no critical incident. Scenario 2 or 3: In a randomized order, workload was increased by simulation of a critical incident | Pupillometry: Pupil size. | Mean pupil diameter increase 8.1 % (SD ± 4.3) in less experienced (<2yrs), 15.8 % (±10.4) in more experienced subjects (>2yrs) (p = 0.191). | Experienced and less experienced anaesthetists performed induction with and wothout a critical scenario. There was no significant difference in pupil size between expereinced and less experienced participants. |
| Germane load | Wu 2020 | Simulation | Simulated robotic tasks. Tasks were performed in the following order: Camera Targeting, Peg Board, Ring and Rail, Sponge Suturing, Dots and Needles, and Tubes. In each task, lower (easier) levels were presented before higher (more difficult) levels. | "Pupillometrics: size Gaze: entropy Fixation: duration Eyelid metrics: PERCLOS" | Increased CWL corresponded to increase pupil diameter (P<.05), and increased Gaze Entropy (Camera Targeting P=.004, Ring and Rail P<.001, and Suture Sponge <.001). There was no significant correlation with fixation and PERCLOS. | Gaze entropy increased as perceived workload increased, with a correlation of .51. Pupil diameter and gaze entropy distinguished differences in workload between task difficulty levels, and both metrics increased as task level difficulty increased. |
| Germane load | Tien 2015 | Real life | Open inguinal hernia repair | "Fixation: frequency AOI: dwell time pupillometry: size, change in pupil diameter (rate), pupil entropy" | Higher left pupil size (p = 0.032), left pupil entropy (p = 0.007) and right pupil entropy (p = 0.022) in Juniors, and Greater left pupil rate of change (p = 0.022) in Experts during mesh repair. Larger left pupil entropy (P = 0.007) and right pupil size (P = 0.015) pupil entropy and right maximum pupil size (P = 0.032) , and higher left pupil rate of change (P = 0.046) in Experts during closure. | Experts demonstrated higher fixation frequency (P = 0.006) and dwell time on the operative site during application of mesh (P = 0.028), closure of the external oblique (P = 0.003 and P = 0.032) and dwelled more on the sterile field during cutting of mesh (P = 0.019). Nevertheless, NASA TLX scores indicated experts found the procedure less mentally demanding than juniors (P = 0.038). |
| Germane load | Wu 2021 | Simulation | Robotic tasks: Camera Targeting (CT), Peg Board (PB), Ring and Rail (RR), Suture Sponge (SS), Dots and Needles (DN), and Tubes (T) | "Pupillometry: Mean Pupil diameter Gaze entropy" | Change in gaze entropy (rrm = − .37, p<.001) correlated with change in performance (with medium effect size). i.e large increase in performance accompanied by larger decrease in engagement index and gaze entropy, while pupil diameter not correlated with performance. | Aim to measure changes in cogntive and behavioural states as trainees progressed through robotic training program. EEG and pupil diameter a engagement index and mental workload. Changes in performance correlated with changes in EI (rrm = − .25, p < .001) and gaze entropy (rrm = − .37, p < .001). |
| Germane load | Zheng 2012 | Simulation | Objective measures were taken form participants while performing simulated laparoscopic cholecystectomy | Eyelid metrics: Blink rate, Blink duration | "Correlation coefficients (Pearson test) between NASA TLX and blink frequency was –0.17 (P = 0.282) and blink duration 0.446 (P = 0.776). " | Eye tracking of surgeons while performing virtual laparsocopic cholesystectomy. Reduced blink freqeuncy and blink duration associated with increasing mental workload. Interestingly, surgical task performance was not associated with the blink frequency of the surgeons. |
| germane load | Richstone 2010 | Simulation and Real life | Simulated tasks of varying degrees (easy, moderate, difficult), and Live task consisted of mobilsation of colon | "- Indexed of Cognitive Activity (ICA) assessed through the following 7 variables: blink rate (left and right eye), fixation rate (left and right eye), pupil metric (left and right eye), and vergence. - Average fixation rate" | "In the simulation study, the mean ICA values for left and right eyes for experts were much lower than those of the nonexperts, with expert values of 1.56 and 2.44 compared with nonexpert values of 4.59 and 3.31. In live surgeries, expert values of 6.05 and 2.85 for left and right eyes, ie lower than nonexpert values of 11.79 and 9.57. For the simulator study, the average fixation rate over both eyes for the experts was 80% compared with the nonexpert fixation rate of 66%; In live surgery, the rates for experts and nonexperts were 51% and 42%. " | Used linear discriminate analysis (LDA) and nonlinear neural network analyses (NNA) to classify surgeons into expert and nonexpert cohorts, based on the relationship between complex eye and pupillary movements, collectively referred to as eye metrics, and surgical skill level. Twenty-one surgeons participated in the simulated and live surgical environments. In the simulated surgical setting, LDA and NNA were able to correctly classify surgeons as expert or nonexpert with 91.9% and 92.9% accuracy, respectively. In the live operating room setting, LDA and NNA were able to correctly classify surgeons as expert or nonexpert with 81.0% and 90.7% accuracy, respectively. |
| Germane Load | Anschueutz 2019 | Simulation | Cadaveric tympanoplasty and stapedectomy in EES in residents and consultants | Fixation duration | Residents had longer fixation duration in the stapedotomy task than in the tympanoplasty task, independent of the endoscopic technique used (mean change, -0.24 seconds; 95% CI, -0.36 to -0.12 seconds). | Trainee and expert surgeons performed ESS under 2D vs 3D view. in relation to the difference between Trainees and Experts, Residents had higher fixation duration indicating higher CWL. |
| Intrinsic load | Diaz-Piedra 2017 | Simulation | Simulatied stone retrieval | Gaze entropy and velocity | "M (SD): 4.71 (0.66) vs 5.04 (0.67) P<0.5 M (SD): 0.28 (0.20) vs 0.35 (0.23) P<0.5" | Gaze entropy and velocity were significantly higher when surgeons performed the more complex surgical procedure: the visual exploration pattern became less stereotyped (ie, more random) and faster. The extraction of a stone in the lumbar right ureter (complex) induced higher gaze dispersion, t (14) = 2.33, P = .035, and higher gaze velocities, t(14) = 2.19, P = .045, when compared to the extraction of a stone in the bladder (simple). Gaze entropy (bits) during bladder stone extraction =4.71(0.66) {mean(SD)}, during ureter stone extraction =5.04 (0.67). Gaze velocity (*/s) during bladder stone extraction = 0.28(0.20), during ureter extraction =0.35(0.23). Surgeons reported higher levels of perceived complexity during the extraction of a stone in the lumbar right ureter (NASA- TLXLumbar Ureter Stone [M ± SD] = 60.78 ± 17.11 vs NASA- TLXBladder Stone [M ± SD] = 54.83 ± 15.95) |
| Intrinsic load | Di Stasi 2016 | Simulation | Objective measures were taken form participants while performing simulated laparoscopic tasks of different complexity (Complexity: Low (Clip Applying); Medium (Cutting Big); High (Translocation of objects)) | "Gaze entropy Gaze velocity " | "M ± SD: 6.798 ± 0.7 vs 6.939 ± 0.9 vs 7.654 ± 0.7; P<0.05 M ± SD: 6.754 ± 0.1 vs 7.472 ± 0.1 vs 8.797 ± 0.1; P<0.05" | Gaze entropy and velocity linearly increased with increased task complexity: Visual exploration pattern became less stereotyped (i.e., more random) and faster during the more complex exercises. Residents performed better the Clip Applying exercise and the Cutting Big exercise than the Translocation of Objects exercise and their perceived task complexity differed accordingly |
| Intrinsic load | Jiang 2013 | Simulation | Objective measures were taken form participants while performing real life laparoscopic tasks:"Peg transfer,reaching and grasping the object (RG), transporting and releasing the object (TR), and bringing the instrument to the home position in the white central square (H)." | "Avergae adjusted pupil diameter Average rate of pupil diameter change *" | "No significant difference (ANOVA F(2,33) = 1.553, p = 0.227) Higher CWL causes rapid pupil dilatation (ANOVA F(2,33) = 41.837, p < 0.001)" | pupil size responds to changes in difficulty, not just to changes in target size. This implies that pupil diameter can be employed as an indicator of task requirement in goal-directed movements, because higher task difficulty evoked higher peak pupil dilation which occurred with longer delay |
| Intrinsic load | Jiang 2014 | Simulation | Objective measures were taken form participants while performing real life laparoscopic tasks: 1) aiming at targets with 4 different sizes located at constant distance apart, and 2) aiming at targets varying in 4 different distances. | Pupil dilation | (data are in graph form in this paper) Linear regression of mean peak pupil dilation for each ID (index of difficulty) to ID value is significant, with R2 = 0.849 and p < .005. Linear regression of mean peak pupil dilation duration (from tooltip-leave) for each ID to ID value is significant, with R2 =0.662 and p < .05. | 8 participants (students) performed simulated laparoscopic tasks. Change in pupil size was assessed in response to different target distance, target sizes and task difficulty. Results showed that the pupil responds to the changes of task difficulty, not just to the change of target size. |
| Intrinsic load | Schulz 2011 | Simulation | "clinical scenarios (x3) during injuction. Scenario 1: familiarisation; no critical incident. Scenario 2 or 3: In a randomized order, workload was increased by simulation of a critical incident " | "Mean Pupil diameter % change in pupil diameter Fixation duration Saccade amplitude Mean HR" | "Weighted Mean pupil diameter did not differ significantly between uneventful and incident sessions P=0.136 Change of pupil diameter %: The mixed-models assessments showed a global development of high statistical significance (P=0.001). An overall assessment of duration of fixation as a function of simulator state by mixed models revealed a highly significant association (P=0.001) In mixed models, Saccade Amplitude did not significantly correlate with the severity of anaphylaxis (P=0.09, data not shown). Mean HR mixed model is highly significant (P=0.001). " | Eye tracking and heart rate of 15 anaesthetic trainees recorded during simualtion with varying levels of workload. Pupil side and HR reflected workload within simulator sessions. Contrary to hypthesis, fixation durtation decreased with increased workload and saccade amplitude did not reflect workload. |
| Intrinsic load | Zhang 2021 | Simulation | Objective measures were taken form participants while performing simulated laparoscopic tasks in 2D and 3D (Peg transfer, Precise positioning, Stitching, Pattern cutting) | "Average fixation rate Saccade speed " | "Greater average fixation rate (p-values = 0.001, 0.000, 0.003 and 0.015, respectively) in 3D Faster saccade speed (p-values = 0.037, 0.003, 0.073 and 0.105, respectively) in 3D" | Eye metrics of surgeons measured while performing simulated laparoscopic tasks in 2D and 3D environments. Greater average fixation rate and saccade speed in 3D over 2D. Lower workload in 3D. |
| Intrinsic load | Zheng 2015 | Simulation | Objective measures were taken form participants while performing simulated laparoscopic task (transport a rubber object (4.5 mm × 10.5 mm green cylinder) over 3 dishes (13 mm in diameter) in a predetermined order). The task can be divided into 9 steps of 3 types of subtask: reaching and grasping (RG, estimated ID = 4.0 bits/response), transporting and releasing (TR, estimated ID = 3.3 bits/response), and homing (H, estimated ID = 2.6 bits/response). | Pupil dilation | Pupil responses were significantly different between 3 subtasks during the fast moving phase, but not between RG (reaching and grasping) and TR (transporting and releasing) during the slow aiming phase. (see chart) | Participants undertook a laparoscopic task divided into 9 subtasks (divided into 3 levels of difficulty). Peak pupil size increased with task difficulty. Results showed that pupil responses were significantly different between 3 subtasks during the fast moving phase, but not between RG and TR during the slow aiming phase. As the entire procedure was performed continuously, we found that pupil responses were not only affected by the ID in the current subtask but also influenced by subtasks before and after. |
| Intrinsic load | Yang 2022 | Simulation | Partcicpants performed RAS intracorporal suturing under High (haemorrhage condition + extrenous noise) and Low MWL (non-haemorrhage). | "Nearest neighbouring index (NNI) Scan path length Average Fixation count Average Fixation duration" | "Scan path length (F (1, 22) = 7.04; p = 0.03) and NNI (F (1, 22) = 18.82; p < 0.001) were 0.58 and 0.04 higher, respectively, in the hemorrhage condition than in the non-hemorrhage condition. Average fixation count not significant Average fixation duration not significant" | The physiological differences between the two task conditions suggest that the designed high MWL hemorrhaging condition evoked high MWL. In the hemorrhage case, longer scan path (i.e., dispersed fixations) and higher NNI (i.e., random fixations) during visual exploration have been associated with higher MWL |
| Intrinsic Load | Anschueutz 2019 | Simulation | Cadaveric tympanoplasty and stapedectomy using 2D versus 3D endoscopy in EES in residents and consultants | Blink rate | Blink rate ↑in 3D endoscopy (mean [SD], 9.10 [8.45]) > 2D endoscopy (6.68 [5.40]), independent of task/experience (mean [SD] change, 2.42 [1.02]; 95% CI,0.26-4.57; r = 0.49). Fixation duration: for residents, ↑ in stapedotomy > tympanoplasty independent of technique (mean change, -0.24 seconds; 95% CI, -0.36 to -0.12 seconds). Experts:↑fixation 2D (0.79 s) vs 3D endoscopy (0.54 seconds). | "Trainee and expert surgeons performed ESS under 2D vs 3D view. Objective measures were time taken to perform the EES, number of attempts, accidental damage during the dissections; eye tracking. Subjective measures were cognitive load and subjective feedback questionnaires. Time to perform was 181 (2D) vs 187 (3D), surgeons preferred 3D (10 for 3D vs 6 for 2D). Eye strain was higher in 3D on 7-point likert scale(3D, 2.19 points vs 2D, 1.44 points; mean difference , 0.74; 95% CI, 0.29-1.20; r = 0.67). Eye movement assessment revealed a higher duration of fixation for consultants in 2D (0.79 seconds) compared with 3D endoscopy (0.54 seconds). Residents (mean [SD], 49.02 [16.4]) had a significantly higher workload than consultants (mean [SD], 27.21 [12.20]), independent of the used technique or task. " |
| Germane Load + Intrinsic | Zhang 2018 | Simulation | Objective measures were taken form participants while performing simulated laparoscopic (peg transfer, clipping, fine separation, vascular shear) | "Saccadic velocity (SV) Mean blink rate (MBR) Mean pupil diameter change (MPDC) fixation time rate (FTR)" | "PT: 95.54 ± 3.17, CS:93.19 ± 9.12, FS: 94.33 ± 4.61, VS: 90.03 ± 17.31 PT: 2.94 ± 3.73 , CS: 2.07 ± 1.47, FS: 4.71 ± 5.78, VS:4.35 ± 5.67 PT: 0.22 ± 0.08, CS: 0.24 ± 0.08, FS: 0.26 ± 0.1, VS: 0.24 ± 0.08 PT: 80.58 ± 43.07, CS: 88.61 ± 48.01, FS: 106.56 ± 60.74, VS: 95.54 ± 3.17 " | The aim of the study was to determine whether eye metrics can identify the "mental workload state"of surgeons. Sixteen surgeons and medical students were recruited to complete four laparoscopic procedures. Eye movement was recorded during the tasks, and NASA-TLX scales were used. There were significant differences in the mental workload of each task, and a significant correlations between mean pupil diameter change and NASA-TLX scores. |
| Germane Load + Intrinsic | Distasi 2017 | Simulation | Objective measures were taken form participants (trainees and experts) while performing complex task (Peg Transfer) and low complexity task (Pattern Cut) using single site (LESS) vs. multiport laparoscopic surgery (MPS) | "Gaze entropy Gaze velocity " | "Gaze entropy higher for LESS compared to MPS in both novices and experts; p=0.011. Gaze velocity had no correlation with CWL in LESS vs MPS or with task complexity Gaze velocity higer for peg transfer than Pattern Cut in MPS (0.383 vs 0.354; P=0.009). " | Surgeons and trainees performed two exercises (low complexity and high complexity) LESS and multiport laparoscopic surgery. NASA-TLX was higher for LESS compared to MPS. This finidng was mirrored by Gaze Entropy, but not Gaze Velocity. NASA-TLX was higher in trainees compared to attending surgeons (experts) indicating higher CWL; however, there was no siginificant difference in Gaze metrics. |
| Germane Load + Intrinsic | Berguer 2001 | Simulation | "Objective measures were taken form participants while performing three 2 minute tasks (1. Rest, 2. Open knot tying, 3. Lap knots) " | Blink rate | No effect size reported: Eye blink rate decreased from rest to the open task (p <0.01) and then increased from the open to the VES task (p< 0.01), though it did not return to the rest level. There were no differences in the results for the subjective ratings or the eye blink rate based on the experience level of the subjects | Surgeons perfomed a knot tying task under open conditions or videoendoscopic conditions, the measures were subjective reports of stress and concentration ("workload"), eye movements (blink rate and fixation duration), and task performance (amount of knots tied). |
| Germane Load + Intrinsic | Dalveren, G 2017 | Simulation | Dominant vs non-dominant vs both hands | Pupil dilation |  |  |
| Germane Load + Intrinsic | Dalveren.G 2018 | Simulation | Hand dominancy and various scenario fideliti | Fixation rate and duration |  |  |
| Germane Load + Intrinsic | Dalveren 2018 (ECE) | Simulation | Handominancy | Pupil dilation |  |  |
| Germane Load + Intrinsic | Gunawardena 2019 (1) | Real life | Objective measures were taken form participants while performing real life laparoscopic cholecystectomy | "Number of fixations per 20 seconds * Fixation rate within one second time interval * Mean fixation duration within 20 seconds Average pupil diameter Mean Percentage Change in Pupil Diameter (PCPD) Index of Pupillary Activity (IPA)*" | "Experts have smaller number of fixations (ANOVA F1,37 = 32.55,p < 0.001) No main effect for fixation rate (ANOVA F1,37 = 0.009,p = 0.926) Experts had a significantly longer fixation duration (ANOVA F1,37 = 18.59,p < 0.001) Pupil diameter is not significantly depending on the surgeons’ expert level (ANOVA F3,35 = 1.152,p = 0.29) No significant difference of PCPD (ANOVA F1,37 = 2.288,p = 0.138). Experts have lower Mean IPA values (ANOVA F1,37 = 13.02,p = 0.0011) " | "4 participants (novice, semi-expert and expert) performed laparascopic cholecystectomy. The results showe that experts (low CWL) have smaller number of fixations (ANOVA F1,37 = 32.55,p < 0.001), longer fixation duration (ANOVA F1,37 = 18.59,p < 0.001), and lower Mean IPA values (ANOVA F1,37 = 13.02,p = 0.0011). There was no signifcant diference in fixation rate (ANOVA F1,37 = 0.009,p = 0.926), Average Pupil diameter (ANOVA F3,35 = 1.152,p = 0.29), or PCPD (ANOVA F1,37 = 2.288,p = 0.138) between experts and non experts " |
| Germane Load + Extraneous | Gao 2018 | Simulation | Objective measures were taken form participants while performing simulated Laparoscopic appendicectomy in noisy room vs silent vs music. | Change in Pupil size | "Pupil dialation was greater under Noise than under silent or music (P<0.0001). " | "Participants (experts and less experienced) performed simulated lap procedure under Noise, Silent and Music Conditions. Fore extrenous load, NASA-TLX showed that CWL was higher under noise conditions (P=0.03) than under silent conditions, and that music conditions led to lower scores (P > 0.05) that did not differ from those generated under silent conditions. Pupil dilation was greater under noise conditions than under Silent and Music and had a high degree of variance (P < 0.0001). Additionally, mean changes in pupil diameters were small and varied little under music conditions and did not differ significantly with those measured in silence (P > 0.05), which mirrors the results reported by NASA-TLX. " |

**Supplemental Table 8: Summary of EEG and fNIRS studies**

| CLT Domain | Study author, year | Study Population | Task | Device | Region of Interest | Data analysis | Objective Parameter | Multimodal | Summary Notes |
| --- | --- | --- | --- | --- | --- | --- | --- | --- | --- |
| Germane | Leff 2008 | Surgery (n=62); 19 consultants, 21 registrars, 22 medical students | Block design: 5 hand-tied knots | Hitachi ETG-4000 Optical Topography System (24 optodes) | Entire PFC (across all 24 optodes) | POOLED & BY CHANNEL: Averaged across all 24 channels; By channel (Friedman analysis for ∆HbO2 and ∆HHb across trials ; WIlcoxon of ∆HbO2 and ∆HHb by intervals) | " Averaged across 24 channels for novices: ↑∆HbO2 coupled to↓∆HHb from Knot 1 to knot 2 (p≤0.01) ↓∆HbO2, ↑∆HHb with knot3 to knot 4 and 4 to 5 (p≤0.01) " | ─ | ─ |
| Germane | Crewther 2016 | Surgery (n=12; 1 excluded) | 3x 3 block design intracorporal laparoscopic suturing | Hitachi ETG-4000 Optical Topography System (44 channels); Zephyr Bioharness 2.0 | 44 channels recording at 8Hz; 24 overlying PFC. Left PFC and Right PRC. | POOLED: Optode ∆HbO2 pooled across all LS tasks for single-session value for left and right PFC. Laterality Index ((right-left)/(right+left) | L & R PFC activity ↑in all sessions (p<0.035); main session effect on PFC (χ2 (3) = 8.13, p=0.043) but no significant session effect on post-hoc analysis | ECG (HR, HRV) - session effects on HF (χ2 (3) = 13.3, p=0.004); HRV p<0.0009; LF (χ2 (3) = , p=0.009; salivary testosterone & cortisol (no significant session differences) |  |
| Germane | Nemani 2018 | Surgery (n=30); experts v novice (med students) | Block design: 5x Timed FLS pattern-cutting task with gauze | 32-channel, near- infrared spectrometer for this study, which delivered infrared light at 690 and 830 nm (CW6 system, TechEn Inc.). The system used eight long-distance and eight short-distance illumination fibers coupled to 16 detectors. | PFC, M1, SMA (left PFC (source 1, detectors 1 and 2), medial PFC (source 2, detectors 2 and 3), right PFC (source 3, detectors 3 and 4), left lateral M1 (source 4, detectors 5 to 8), LMM1 (source 5, detectors 8 to 10), right medial M1 (source 6, detectors 9 to 12), right lateral M1 (source 7, detectors 11 to 14), and finally, SMA (source 8, detectors 9, 15, and 16).) | ROI: ∆HbO2 and ∆HHb; linear discriminant analysis (LDA) to estimate misclassification errors (MCEs) | Sig diff ∆HbO2 observed in all PFC, SMA, lt medial M1 (LMM1), and the rit lateral M1 regions. Novices sig. ↑functional activation in the PFC regions (P < 0.05) and sig ↓ functional activation LMM1 & SMA vs experts. Poor intragroup MCEs for the Unskilled trainee population (MCE12 24% and MCE21 47%). In contrast, we completely classified Skilled trainees on the final training day from Skilled trainees on the pretest, with MCE12 0% and MCE21 0% | _ |  |
| Germane | Walia 2022 | Surgery (n=22); med students vs attending/residents >1 lap yr | Block design: x3 Intracorporal knot tying through penrose drain slits to oppose | "fNIRS: 32-channel fNIRS signals & 8-channel short-separation channels 5 Hz sampling rate with NIRSPORT2; EEG: 32-channel wireless LiveAmp system (Brain Vision, USA" | "Ventrolateral prefrontal cortex (VFC), superior parietal lobule (SPL), supramarginal gyrus (SMG), angular gyrus (AG), dorsolateral prefrontal cortex (DLPFC), frontal eye feld (FEF), premotor and primary motor cortex (PMC)" | BY CHANNEL & ROI(indiv channels): ΔHbO2; EEG signal ->microstate analysis (6 microstate prototypes explained 77% of global variance. GLM classification of error-related events. ANOVA of expert v novice | "Six EEG microstate prototypes explained 77.14% of the global variance, % total time in diff microstates sigby the skill level (p<0.01), the microstate type (p<0.01), and the interaction b/w skill level*microstate type (p<0.01). fNIRS: HbO Lt inferior frontal gyrus(opercular part), lt superior frontal gyrus(medial orbital), lt postcentral gyrus, lt superior temporal gyrus, rt superior frontal gyrus (medial orbital) all (p<0.05) diference between experts and novices in the 10-s error epoch" | Yes: fNIRS (ΔHbO2) and EEG microstates |  |
| Germane | Modi 2018 | Surgery (n=33); Surgical residents (Novices, intermediate and experts) | Block design: 5 Laparascopic suturing knots under Time Pressure and Self Paced conditions (between group comparison) | ETG-4000 Optical Topography System (Hitachi Medical Co, Japan) 24 optodes; ; Zephyr Bioharness 2.0 | Entire PFC (across all 24 optodes) | BY CHANNEL & ROI: ∆Hb (Hb task- Hb rest) was compared for each channel using the Wilcoxon, and for subgroups using the Kruskal-Wallis followed by Post Hoc Mann-Whitney U. Random effects model for the influence of expertise of TP on ∆HbO2 and ∆HHb. Optodes for ROI not specified; in diagram form | Random effect analysis: expertise was not a predictor for DHbO2 (z =-0.59, P = 0.557) or DHHb (z =-1.83, P =- 0.068). |  |  |
| Extraneous |  |  | Block design: 5 Lap suturing knots under Time Pressure and Self Paced conditions (within group comparison) |  |  |  | Random effect analysis: SP vs TP was not a predictor of DHbO2 (z =-1.17, P = 0.244) or DHHb (z =- 1.58, P = 0.113). | No significance in ∆HR SP vs TP |  |
| Extraneous | Modi 2019 | Surgery (n=33); Surgical residents. Technical performance => composite deterioration score. Q1 vs Q4 | Block design: 5 Lap suturing knots under Time Pressure and Self Paced conditions (within group comparison) | "ETG-4000 Optical Topography System (Hitachi Medical Co, Japan) 24 optodes; ; Zephyr Bioharness 2.0 " | "Entire PFC (across all 24 optodes) " | BY CHANNEL & ROI: Normalised ∆performance score %weight to give composite degradation score (Q1 stable vs Q4 decline). ΔHbO2 Q1 vs Q4 independent-samples t test | Q1 task-induced↑∆HbO2 B/L VLPFC& right DLPFC in the SP condition and in the VLPFC in the TP condition; Q4 ↓HbO2 in both SP and TP. ∆HbO2 sig Q1>Q4 B/L VLPFC during both SP (mean [SD] left VLPFC: Q1, 0.44 [1.30] μM; Q4, −0.21 [2.05] μM; P <0.001; right VLPFC: Q1, 0.46 [1.12] μM; Q4, −0.15 [2.14] μM; P < .001) and TP (mean [SD] left VLPFC: Q1, 0.44 [1.36] μM; Q4, −0.03 [1.83] μM; P = .001; right VLPFC: Q1, 0.49 [1.70] μM; Q4, −0.32 [2.00] μM; P < .001) | Yes: fNIRS (ΔHbO2) and median HR; No significance in ∆HR Q1 v Q4 |  |
| Extraneous | Modi 2020 | Surgery (n=29); all residents | "Block design:Particpants performed 5 intracorporial laparoscopic suturing on a box trainer tasks under two conditions: self-paced (SP) and Time-Pressure (TP). For each condition, participants tied 5 interrupted knots with a 30-second intertrial rest period. " | ETG-4000 Optical Topography System (Hitachi Medical Co, Japan) 24 optodes; ; Zephyr Bioharness 2.0 | "Entire PFC (across all 24 optodes) " | "BY CHANNEL & ROI(indiv channels): ∆Hb (Hb task- Hb rest) was compared for each channel using the Wilcoxon, and for subgroups using the Kruskal-Wallis followed by Post Hoc Mann-Whitney U. Random effects model for the influence of expertise of TP on ∆HbO2 and ∆HHb. " | (∆ HbO2) was significantly less (P < 0.05) in the bilateral DLPFC and VLPFC with dual task. Random effects analysis showed significant decrease in ∆ HbO2 in the bilateral DLPFC and VLPFC with dual task, but not TP. | No significance in ∆HR |  |
| Extraneous | Singh 2018 | Surgery (n=8) ; 1 consultant, 7 trainees | Block design: Lap suturing (self-paced v time-pressured); robotic (self paced v time-pressured) after month washout | ETG-4000 Optical Topography System (Hitachi Medical Co, Japan) 24 optodes; ; Zephyr Bioharness 2.0 | Entire PFC (across all 24 optodes) | CHANNEL & ROI: Optode ∆HbO2 & ∆HHb; analysis of both 24 independent channels and region of interest (e.g. VL PFC, DLPFC) | "SP: 20/24 task-induced ↑∆HbO2 and ↓∆HHb (Ch 20 & 21 sig for both ↑∆HbO2 and ↓∆HHb) TP: 20/24 task-induced ↑∆HbO2 (CH 6 sig) and 15/24 ↓∆HHb (6 Ch sig). Attenuated responses ∆HbO2 in TP compared to SP in 13 Ch (B/L VLPFC and DLPFC)" | LS: No significance in ∆HR SP vs TP | "Only 4/768 channels sig (P<0.05) correlations (R>0.8) between HR and significant ∆HbO2; SP: No sig. correlations b/w HR and ∆HbO2 in LS; 1 channel (19) in 1 pt for RS TP: 2 channels (9 &21 in 1 pt) in LS; 1 channel (4 in 1 pt) in RS" |
| Intrinsic |  |  |  |  |  |  | "SP: smaller ∆HbO2 response robotic <lap in 21 channels (did not reach significance); TP: greater ∆HbO2 response robotic <lap in 7 channels (right VLPFC)" | SP: No significance in ∆HR |  |
| Intrinsic (task difficulty) | Izzetoglu | Surgery (n=24); All residents | Block design: each participant completed two blocks of sponge suturing tasks. Block 1 presented in difficulty order, while block 2 presented the tasks randomly. | Imager 1200 (fNIR Devices LLC., Potomac, MD, USA) 16 optodes | Left and Right DLPFC AMPFC | ROI AND BY CHANNEL (optodes 3&4): ∆HbO and ∆HbR (deoxy) and OXY (HbO-HbR). Left DLPFC (optodes 1-4); left AMPFC (optodes 5-8); Right AM PFC (optodes 9-12); Right DLPFC (optodes 13-16) | The main effect of task difficulty was significant primarily in left DLPFC |  |  |
| Intrinsic (task difficulty) | Shugaba 2023 | Consultant LS and RS surgeons: General Surgery (Colorectal); Urology; Gynaecology (n=13 (7 LS; 6 RS)) | (1) noncritical bowel dissection, (2) critical vessel dissection, and (3) dissection after vessel control in LS (14 colectomies, 12 hysterectomies) and RS (19 nephrectomies, 9 colectomies) | Enobio 8 5G wireless device (Neuroelectrics, Cambridge, MA) was used to record EEG data from 8 channels | channels (Cz, Fz, P7, P8, P3, P4, O1, and O2) | Power spectral density (PSD) in alpha band for cognitive demand; beta for fatigue; alpha event related desynchronisation | Sig. effect surgery time on the EEG activity (P < 0.0001). ↑ cognitive demand RS > LS (alpha, beta, theta, delta, and gamma (P = 0.002 – P <0.0001)). Alpha event-related desynchronization sig RS>LS . Mixed-model ANOVA indicated a sig effect of modality on alpha power, whereby alpha power was significantly lower during RS relative to LS (P < 0.001), indicating overall greater attentional demand in RS relative to LS | _ |  |
| Intrinsic (task difficulty) | Morales 2019 | General Surgery (n=8) | 2x2x2 design live lap porcine (continuous v interrupted; single v multiport; primary v assistant) | NeuroSky Mindwave Mobile; 1 electrode (Fp1) | Frontal | Beta (13-30 Hz) | Task complexity β-activity overall interaction: F(1,7) =8.41, p<0.05. Average values (SD): MPS Contin 2.891 (0.71); MPS interrup. 28.89 (0.76); LESS contin 29.62 (1.81); LESS interrupted 29.00 (1.40) | _ |  |
| Intrinsic (task difficulty) | Plazak 2019 | Novice (Med imaging/surgical technology researchers n=13) | Navigated a surgical tool to a 3D virtual location on CT angiogram. Each ppt task x8 visual feedback only, x8 aural feedback only, x8 visuo-aural feedback. | Muse headband EEG (7 sensors; 4 channels), alpha band relative to theta, beta and gamma bands. Sampling rate 10Hz | Frontal | Repeated measures ANOVA of task accuracy as a function of visual, aural, and visuo-aural stimulus. Repeated measures ANOVA of cognitive load (EEG) as a function of visual, aural, and visuo-aural stimulus. | "EEG: visual vs aural vs visuoaural stimulus -F(2, 12) = 4.24, p = .0154. Task accuracy visual vs aural vs visuoaural stimulus | ANOVA F(2, 12) = 2.894, p = .057;" |  |
| Intrinsic (task difficulty) | Guru 2015 (2) | Urology (n=1 expert) | 21 prostatectomies, 26 cystectomies, 4 reconstructions. All broken down into 3 key portions: A) Lysis of Adhesions; B) eLND; C) UVA | ABM X1 neuro-headset; 20 electrodes | Frontal, central, parietal, occipital sensors | Low & high level engagement, mental state, cognitive load and overall cognitive score extracted from pre-processed signals. Spearman's rank correlation coefficient (2-sided; p<0.05 sig) | LoA: Effort (0.4; p=0.05); eLND WL no sig correlation; UVA: Mental demand (-0.53; p=0.02); Temporal demand (-0.56; p=0.01); Performance (-0.46; p=0.05); Frustration (-0.48; p=0.04) | _ |  |
| Uncontrolled (Live procedure) | Hussein 2016 | Urology (n=1 expert) | 20 procedures broken down into A) eLND and B) UVA | ABM X1 neuro-headset; 20 electrodes (256 samples/sec) | Frontal, temporal, parietal, occipital sensors | Distraction, high level engagement, cognitive state and workload extracted from pre-processed signals. Spearman's rank correlation coefficient (2-sided; p<0.05) | eLND: mental demand r=-0.74 (p=0.05); Effort r=-0.86 (p=0.01); Frustration r=-0.84 (p=0.02). UVA: not sig correlation. | _ |  |
| Intrinsic (task difficulty) | Guru 2015 | Urology (n=10); 2 beginner, 5 proficient, 3 expert | Robotic simulation tasks: Basic (ball placement, suture pass, ring peg trasnfer) v Intermediate (simple suture with knot tying) v Advanced (UVA on model) | ABM X1 neuro-headset; 9 electrodes | Frontal, central, parietal, occipital sensors | Low & high level engagement, mental state, cognitive load and overall cognitive score extracted from pre-processed signals. Wilcoxon rank-sum test (2-sided; p<0.05 sig) | No statistical analysis to compare high vs low intrinsic WL | ─ |  |
| Germane |  |  | (Advanced not performed by novices) |  |  |  | Cognitive load: BASIC: CPG vs EG p = 0.021; INTERMEDIATE: BG vs EG p =0.033 CPG vs EG 0.027; ADVANCED: CPG vs EG 0.024 |  |  |
| Intrinsic (task difficulty) | Maddox 2015 | Urology (n=19): 6 med students; 9 residents; 4 attending | Laparoscopic simulation: Peg transfer v lap suturing | MUSETM headband sensors: 7 electrodes | 2 forehead, 2 ear, 3 reference - prefrontal | Gamma (30-100Hz) and Alpha (7-14Hz) -> composite stress and concentration scores (2-sided; p<0.05) | Tasks not compared statistically | _ |  |
| Germane |  |  | Novice v Intermediate v Expert |  |  |  | Concentration levels sig. better EG>less experiencedc during both the peg and suture tasks (P = 0.036, P = 0.0039) |  |  |
| Germane | Maimon 2022 | Medical student laparoscopy novices (n=38) | Lap simulation: 1) Lap grasping x3; 2) 10 subj repeated 3 on subsequent day; 3) Same task 1/day 3 consecutive days | Aurora by Neurosteer; single channel via 3 forehead electrodes (Fp1, Fp2 and reference Fpz) | Frontal | "Delta, Theta, Alpha, Beta, Gamma and VC9 (machine-learning-based cognitive load biomarker)" | VC9 significantly ↓ trial1->2 (p = 0.011, d = 0.474) and trial 1->3 (p = 0.021, d = 0.503). Delta sig ↓ trial1->2 (p = 0.003, d = 0.625). Theta did not exhibit any significant differences between trials | _ |  |
| Intrinsic (task difficulty) | Shafiei 2016 | n=1 urologist | Expert surgeon watched 3 trainees performing a total of 87 urethrovesical anastomosis UVA (simple task) and 83 radical prostatectomy with lymph node dissection LND (complex task) | ABM X-24 neuro-headset. EEG data from each channel was sampled at 256 samples per second.12 cognitive measures and 21 functional measures extracted from EEG theta, alpha, beta and gamma placed in expert's head.(Expert completed NASA-TLX after each operation to classify trainee performance (trustworthy vs untrustworthy) | Frontal (F), temporal (T), parietal (Pa), central (C), and occipital (O) cortices. | support vector machine with kernel target alignment used for Machine learning classification. | classification accuracy duuring uVA= 98.81% calculated with LOOCV algorithm . accuracy during LND = 98.79% using LOOCV algorithm |  |  |
|  | Suarez Revelo 2019 | n=8 bioengineering students | bimanual coordination task (1 task only) on laparoscopic trainer. 4 sessions, one per week, with task repeated 3 times in each session. | EEG (Natus Quantum) recordings of theta, alpha and beta bands and NASA TLX. EEG Sampling frequency 1024 Hz, 64 electrode cap. | frontal, "posterior"regions | ANOVA of effect of training sessions on 1) task score, 2) NASA TLX, 3) frontal theta 4) posterior alpha 5) central beta | no analysis done of correlation of EEG power indices with task performance or workload index. frontal theta: F (3,21) = 3.243; p =0.043; ɳ2 = 0.317, significant increase first to second session. posterior alpha: F (3,21) = 5.790; p = 0.005; ɳ2=0.453. significant increase first to fourth session. central beta:F (3,21) = 4.230; p =0.017; ɳ2 = 0.377. significant decrease first to fourth session. total task score: F (3,21) = 12.454; p = 0.000; ɳ2=0.640. SIgnificant increase first to second and first to fourth sessions, |  |  |
|  | Wu 2021 | n=7 urology trainees | simulated robotics skills exercise . 12 exercises performed at each training session, total 26 training sessions over 3 months | EG EMOTIV Epoch at 128 Hz on 14 channels+ gaze tracking system Tobii pro glasses 2.0 | Not specified | Alpha, beta, theta. Separate ANOVA models for each of pupil diameter, EI, and gaze entropy for improvement vs no improvement group. ANOVA of NASA scores for pupil diameter and engagement . Repeated measures correlation. Machine learning classification using logistic regression, Naive Bayes, and support Vector Machine. | EEG: EI = beta /(alpha + theta) using posterior channels P7 and P8 on EEG.Retrieving data. ANOVA: F1,120 = 10.02,p = .002. (mean values represented graphically) |  | Improvement vs no improvement |
| Intrinsic (task difficulty) | Yang 2022 | n=10 novice robotic surgeons | RAS intracorporal suturing: non-haemorrhage vs haemorrhage condition | 32 channel EEG Nautilus EEG at 250Hz | Not specified | Theta (4–8 Hz), alpha (8–13 Hz), and beta bands (13–30 Hz). Band power was averaged across all available channels. Repeated measures ANOVA. NN | EEG theta (F (1, 22) = 10.25; p = 0.04) and beta (F (1, 22) = 16.45; p = 0.03) band powers of EEG was 4.69 and 4.24 higher in hemorrhage condition when compared with non-hemorrhage condition no significant differences were observed in alpha | "Modality Accuracy Sensitivity Precision Multi-sensor 77.9% ± 5.9% 82.4% ± 8.9% 75.7% ± 6.5% EEG only 63.4% ± 6.2% 64.5% ± 10.5% 62.3% ± 13.4% Eye-tracker only 70.1% ± 11.5% 79.4% ± 7.8% 62.8% ± 12.6%" | "The significant increases in theta and beta activity can also provide evidence of the high MWL evoked in the hemorrhage condition. The increased theta and beta in hemorrhage conditions suggested that more cognitive resources were needed to accomplish the task. No differences were detected in alpha, fixation duration, and fixation count" |

**Supplemental Table 9**: Study characteristics by modality (those involving single modality: eye, cardiac, EEG, fNIRS, and multimodal studies). Number and percentage of studies (a) clinical setting of task (i.e. simulated task or in real-life clinical environment; (b) specialty of study; (c) whether the studies defined CWL fully, partially or not at all; (d) subjective assessment tools utilized; and e) Newcastle Ottawa Quality Assessment outcomes

| Modality | Studies (n); | Clinical setting  (n (%)) | Specialty  (n (%)) | CWL Definition (n) | Subjective CWL tool  (n (%)) | Newcastle Ottawa Assessment |
| --- | --- | --- | --- | --- | --- | --- |
| Eye Only | 27 | Sim: 23 (85.2%)  Real-life: 4 (14.8%) | Surgery: 27 (100%) | Defined: 5 (18.5%)  Partial: 8 (29.6%)  Nil: 14 (51.9%) | NASA-TLX: 10 (37.0%)  SURG-TLX: 2 (7.4%)  BORG: 1 (3.7%)  Other: 1 (3.7%) | Good: 26 (96.3%)  Fair: 1 (3.7%) |
| Cardiac Only | 10 | Sim: 2 (20%)  Real-life: 8(80%) | Surgery: 7 (70%)  Anaesthesia: 2(20%)  Interventional Radiology: 1 (10%) | Defined: 2 (20%)  Partial: 2 (20%)  Nil: 6 (60%) | NASA-TLX: 3 (30%)  SURG-TLX: 3 (30%)  BORG: 2 (20%) | Good: 6 (60%)  Fair: 3 (30%)  Poor: 1  (10%) |
| EEG Only | 10 | Sim: 6 (60%)  Real-life: 4 (40%) | Surgery: 10 (100%) | Defined: 3 (30%)  Partial: 5 (50%)  Nil: 2 (20%) | NASA-TLX: 6 (60%) | Good: 5 (50%)  Fair: 4 (40%)  Poor: 1 (10%) |
| fNIRS  Only | 3 | Sim: 3 (100%) | Surgery: 3 (100%) | Partial: 1 (33.3%)  Nil: 2 (66.6%) | Not used | Good: 3 (100%) |
| Multimodal | 17 | Sim: 12 (70.6%)  Real-life: 5 (29.4%) | Surgery: 15 (88.2%)  Anaesthesia: 2 (11.8%) | Defined: 4 (23.5%)  Partial: 7 (41.2%)  Nil: 6 (35.3%) | NASA-TLX: 7 (41.2%)  SURG-TLX: 6 (35.3%) | Good: 16 (94.1%)  Fair: 1 (5.9%) |

**Supplemental Table** **10**: Distribution of studies by modality, parameter and individual metrics identified

| Modality | Studies (n) | Parameters | Studies (n) | Metrics | Studies (n) |
| --- | --- | --- | --- | --- | --- |
|  |  |  |  |  |  |
| Ocular | 33 | Gaze metrics | 6 | Gaze distance  Gaze Entropy  Gaze velocity | 1  4  4 |
|  |  |  |  |  |  |
|  |  | Fixation | 15 | Fixation number  Fixation duration  Fixation frequency  Fixation ratio  Fixation max duration | 6  8  8  1  1 |
|  |  |  |  |  |  |
|  |  |  |  |  |  |
|  |  | Pupillometry | 22 | Change in pupil diameter (percentage, mean, rate, and not specified)  Pupil size (mean, max, average adjusted, unilateral, bilateral, and not specified)  Pupil entropy | 18  5  4 |
|  |  |  |  |  |  |
|  |  |  |  |  |  |
|  |  | Saccade | 7 | Saccade number  Saccade duration (average, not specified)  Saccade velocity (average, peak)  Saccade Amplitude (max, average, not specified)  Saccade length | 3  3  3  2  1 |
|  |  |  |  |  |  |
|  |  |  |  |  |  |
|  |  | Index | 3 | Index of pupillary activity  Index of cognitive activity | 1  2 |
|  |  | Blink | 10 | Blink rate  PERCLOS | 9  1 |
|  |  |  |  |  |  |
|  |  |  |  |  |  |
|  |  |  |  |  |  |
|  |  | AOI | 5 | Dwell time  Scan path | 3  2 |
| Cardiac | 24 | Heart rate | 18 | Mean HR (Mean, max, min, median, minute by minute, and normalised average) | 18 |
|  |  |  |  |  |  |
|  |  |  |  |  |  |
|  |  | Frequency domain | 9 | LF/HF  LF (power, %, absolute, and not specified)  HF ( power, %, absolute, and not specified)  VLF (absolute, and peak frequency)  HF / (HF+LF) | 8  3  3  2  1 |
|  |  |  |  |  |  |
|  |  |  |  |  |  |
|  |  | Time domain | 4 | SDNN  RMSSD  PNN50  PNN20  AVNN  NN50 | 4  3  3  1  1  1 |
|  |  | Non-linear | 2 | (PeEn, SampEn, ShanEn, D2)  (1V, 2LV, SD1, SD2, CVI, CSI) | 1  1 |
| EEG | 14 | Single | 1 | Beta | 1 |
|  |  | Multiple band frequencies | 3 | Theta (4–8 Hz), alpha (8–13 Hz), and beta bands (13–30 Hz) averaged across channels | 1 |
|  |  |  |  | Frontal theta, posterior alpha, and central beta | 1 |
|  |  |  |  | Power spectral density (PSD) in alpha band for cognitive demand; beta for fatigue; alpha event related desynchronisation | 1 |
|  |  | Composite scores | 3 | Alpha and theta combined to give cognitive load score | 1 |
|  |  |  |  | Alpha relative to beta, theta & gamma | 1 |
|  |  |  |  | Engagement Index = Beta / (Alpha + Theta) | 1 |
|  |  |  |  | Composite stress and concentration scores (using area under curve for alpha and gamma waves/TCT) | 1 |
|  |  | Machine learning | 3 | Theta, Alpha, Beta, Gamma used  Cognitive state scores: 1]Mental workload (MW), mental load (ML), situation awareness (SA), engagement (E), blink rate (BR), asymmetry index (AI).  Support vector mchine classification of trustworthiness | 1 |
|  |  |  |  | VC9 (machine-learning cognitive load biomarker); theta, delta | 1 |
|  |  |  |  | Microstate analysis | 1 |
| fNIRS | 9 | Delta HbO2 pooled | 9 | Pooled entire PFC | 2 |
|  |  | Delta HbO2 RoI indiv channels | 7 | L VL PFC | 7 |
|  |  |  |  | R VL PFC | 7 |
|  |  |  |  | L DL PFC | 2 |
|  |  |  |  | R DL PFC | 5 |
|  |  |  |  | L DM PFC | 5 |
|  |  |  |  | R DM PFC | 2 |
|  |  |  |  | SMA | 3 |
|  |  |  |  | M1 | 2 |
|  |  | Pooled subregions | 3 |  | 3 |
|  |  | Delta HHb | 6 | Pooled | 2 |
|  |  |  |  | Individual channel | 4 |
|  |  | OXY | 1 | (HbO-HbR) | 1 |
|  |  | Laterality Index | 1 | (right-left)/(right+left) | 1 |
| Hormonal | 2 | Salimetrics | 2 | Salivary cortisol | 2 |
| Hormonal  Respiratory | 2  3 | Salimetrics  Blood oxygen | 2  1 | Salivary testesterone | 1 |
|  |  |  |  | Mean SPO1 | 1 |
|  |  | Breathing pattern | 2 | Respiratory rate (mean, max, min, frequency) | 2 |

PERCLOS: The PERcentage of eye CLOSure; HR: Heart Rate; LF: Low Frequenc; HF: High Frequency; VLF: Very Low Frequency; SDNN: standard deviation of NN intervals; RMSSD: root mean square of successive differences between normal heartbeats; PNNx: the proportion of NNx divided by the total number of NN (R-R) intervals; AVNN: Average interval between normal heart beats; NN50: The number of pairs of successive NN (R-R) intervals that differ by more than 50 ms; PeEn: Permutation entropy; SampEn: Sample entropy; ShanEn: Shannon Entropy; 1V: patterns with one variation; 2LV: patterns with two like variations; 1SD: instantaneous beat-to-beat variability; SD2: standard deviation of each point from the y = x + average R–R interval; CVI: cardiac vagal index; CSI: Cardiac Sympathetic Index; PFC: Preforntal Cortex; VL: VentroLateral; DL: DextroLateral; DM: DextroMedial

**Supplemental Table 11:** Inductive analysis of task paradigm to determine a) which CLT domain (i.e., extraneous, intrinsic, germane, or uncontrolled) is responsible for the change in CWL and, b) to determine whether the modality had detected the change in CWL. As such, each modality and metric were assigned a label (True Positive, False Positive, True Negative, and False Negative).

| CLT Domain | Task paradigm | Task | Modality | Identifies change in CWL | subjective | Identifies change in CWL | Study |
| --- | --- | --- | --- | --- | --- | --- | --- |
| Extraneous Load | Ambient noise | operating under noise (CABG) | Cardiac | Yes | - | - | Kennedy-metz 2022 ^3^ |
|  | Ambient temperature | Operating number high thermal conditions (Burn surgery) | Cardiac | Yes | SURG-TLX | Yes | Paljewala 2023 ^4^ |
|  | Time pressure | Laparoscopic suturing | fNIRS | Yes | SURG-TLX | Yes | Modi 2020 ^5^ |
|  | Laparoscopic suturing (Expertise) | Median HR | Cardiac | Yes | SURG-TLX | Yes | Modi 2020 ^5^ |
|  | Time pressure | Laparoscopic suturing | fNIRS | No | SURG-TLX | Yes | Modi 2018 ^6^ |
|  | Laparoscopic suturing | Median HR | Cardiac | No | SURG-TLX | Yes | Modi 2018 ^6^ |
|  | Time pressure | Laparoscopic suturing | fNIRS | Yes | SURG-TLX | Yes | Modi 2019 ^7^ |
|  | Time pressure | Laparoscopic suturing | fNIRS | Yes | SURG-TLX | Yes | Singh 2018 ^8^ |
|  | Time pressure | Laparoscopic knot tying | Cardiac | Yes | SURG-TLX | Yes | Singh 2018 ^8^ |
| Intrinsic Load | Operating modality | SILC vs CLC (Cholecystectomy) | Cardiac | Yes | SURG-TLX | Yes | Abdelrahman 2016 ^9^ |
|  | Operating modality | RC vs CLC (Cholecystectomy) | Cardiac | Yes | - | - | Heemskerk 2014^10^ |
|  | Operating modality | SILS vs Multiple ports suturing | EEG | Yes | NASA-TLX | Yes | Morales 2019 ^11^ |
|  | Operating modality | SILS Vs Multiport general tasks | Eye | Yes | NASA-TLX, Borg | Yes | Distasi 2017 ^12^ |
|  | Operating modality | Conventional Microscope vs 3D Microscope (higher CWL in 3D) | Cardiac | No | SURG-TLX | No | Kelkar 2022 ^13^ |
|  | Operating modality | 2D vs 3D Laparoscopic task (lower in 3D) | Eye | Yes | - | - | Zhang 2021 ^14^ |
|  | Operating modality | Robotic vs Lap suturing | fNIRS | Yes | SURG-TLX | No | Singh 2018 ^8^ |
|  | Operating modality | Lap vs Robotic (General live operations) | EEG | Yes | - | - | Shugaba 2023 ^15^ |
|  | Task complexity | Mastoidectomy and Facial Nerve dissection | Cardiac | Yes | - | - | Dedmon 2019 ^16^ |
|  | Task complexity | Stage of anaesthesia (Induction vs maintenance vs emergence) | Cardiac | Yes | NASA-TLX, Borg | Yes | Martin 2016 ^17^ |
|  | Task complexity | Critical events during induction | Cardiac | Yes | - | - | Schulz 2011 ^18^ |
|  | Task complexity | Critical events during induction | Eye | Yes | - | - | Schulz 2011 ^18^ |
|  | Task complexity | Stage of operation (aneurysm clipping) | Cardiac | Yes | SURG-TLX | Yes | Pimentel 2019 ^19^ |
|  | Task complexity | Performing vs Supervising CABG | Cardiac | Yes | - | - | Song 2009 ^20^ |
|  | Task complexity | Stages of operations (Urology) | EEG | Yes | NASA-TLX | with EEG | Guru 2015 (2) ^21^ |
|  | Task complexity | Stages of operations (Urology) | EEG | Yes | NASA-TLX | Corrlated with EEG | Hussein 2016 ^22^ |
|  | Task complexity | Sim CT angio with visual vs aural vs visuo-oral assistance | EEG | Yes | NASA-TLX | Yes | Plazak 2019 ^23^ |
|  | Task complexity | Watching trainees operate | EEG | Yes | NASA-TLX | Yes | Shafiei 2016 ^24^ |
|  | Task complexity | Robotic suturing - haemorrhage v non-haem | EEG | Yes | - | - | Yang 2022 ^25^ |
|  | Task complexity | Robotic suturing | Eye | Yes | - | - | Yang 2022 ^25^ |
|  | Task complexity | Sim stone retrieval - pathology/anatomy | Eye | Yes | NASA-TLX | Yes | Diaz-Piedra 2017 ^26^ |
|  | Task complexity | General Lap tasks | Eye | Yes | NASA-TLX | Yes | Di Stasi 2016 ^27^ |
|  | Task complexity | General Lap tasks | Eye | Yes | - | - | Jiang 2013 ^28^ |
|  | Task complexity | General Lap tasks | Eye | Yes | - | - | Jiang 2014 ^29^ |
|  | Task complexity | General Lap tasks | Eye | Yes | NASA-TLX | Yes | Zhang 2018 ^30^ |
|  | Task complexity | General Lap tasks of increasing difficulty | Eye | Yes | - | - | Zheng 2015 ^31^ |
|  | Task complexity | General Robotic tasks | EEG | Yes | - | - | Guru 2015 ^32^ |
|  | Task difficulty + Teaching the task | Stage of anaesthesia | Cardiac | Yes | Borg | Yes | Weinger 2004 ^33^ |
| Germane Load | Expertise | Sim TEVAR | Cardiac | Yes | NASA-TLX | Yes | Bakhsh 2018 ^34^ |
|  | Expertise | Real life general operations | Cardiac | No | - | - | Carnevali 2023 ^35^ |
|  | Expertise | Sim cerebral angiography | Cardiac | Yes | NASA-TLX | No | Hinzman 2023 ^36^ |
|  | Expertise | Critical events during induction | Cardiac | No | - | - | Schulz 2014 ^37^ |
|  | Expertise | Stage of anaesthesia | Cardiac | Yes | Borg | Yes | Weinger 2004 ^33^ |
|  | Expertise | General Lap tasks | fNIRS | Yes | - | - | Nemani 2018 ^38^ |
|  | Expertise | Laparoscopic knot tying | fNIRS | Yes | - | - | Walia 2022 ^39^ |
|  | Expertise | Laparoscopic knot tying | EEG | Yes | - | - | Walia 2022 ^39^ |
|  | Expertise | Microscopic suturing | Eye | Yes | - | - | Bednarik 2018 ^40^ |
|  | Expertise | Sim Endoscopic Sinus Surgery | Eye | Yes | - | - | Berges 2022 ^41^ |
|  | Expertise | Sim hip arthroscopy | Eye | Yes | NASA-TLX | No | Cai 2022 ^42^ |
|  | Expertise | Laparoscopic knot tying | Eye | Yes | - | - | Cao 2015 ^43^ |
|  | Expertise | Lap Roux en Y procedure | Eye | Yes | - | - | Erridge 2018 ^44^ |
|  | Expertise | Sim Lap appendicectomy | Eye | No | NASA-TLX | No | Gao 2018 ^45^ |
|  | Expertise | Lap Cholecystectomy | Eye | Yes | - | - | Gunawardena 2019 (1) ^46^ |
|  | Expertise | Microscopic suturing | Eye | Yes | - | - | Koskinen 2020 ^47^ |
|  | Expertise | General Robotic tasks | Eye | Yes | - | - | Nguyen 2020 ^48^ |
|  | Expertise | Sim colon mobilisation | Eye | Yes | - | - | Richstone 2010 ^49^ |
|  | Expertise | Critical events during induction | Eye | No | - | - | Schulz 2014 ^37^ |
|  | Expertise | Inguinal hernia repair | Eye | Yes | NASA-TLX | Yes | Tien 2015 ^50^ |
|  | Expertise | Sim Lap cholecystectomy | Eye | Yes | NASA-TLX | used for correlation | Zheng 2012 ^51^ |
|  | Expertise | General Robotic tasks | EEG | Yes | - | - | Guru 2015 ^32^ |
|  | Expertise | Hand tie | fNIRS | Yes | - | - | Leff 2008 ^52^ |
|  | Training effect | General Lap tasks | Cardiac | No | NASA-TLX | Yes | Yu 2022 ^53^ |
|  | Training effect | General Lap tasks | EEG | Yes | NASA-TLX | Yes | Yu 2022 ^53^ |
|  | Training effect | Laparoscopic suturing | fNIRS | No | NASA-TLX | Yes | Crewther 2016 ^54^ |
|  | Training effect | Laparoscopic suturing | Cardiac | Yes | NASA-TLX | Yes | Crewther 2016 ^54^Crewther 2016 ^54^ |
|  | Training effect | General Lap tasks | EEG | Yes | - | - | Maimon 2022 ^55^ |
|  | Training effect | General Lap tasks | EEG | Yes | NASA-TLX | No | Suarez Revelo 2019 ^56^ |
|  | Training effect | General Robotic tasks | EEG | Yes | NASA-TLX | Yes | Wu 2021^57^ |
|  | Training effect | General Robotic tasks | Eye | Yes | NASA-TLX | Yes | Wu 2021 ^57^ |
|  | Training effect | Robotic suturing task | Eye | Yes | - | - | Dilley 2020 ^58^ |
|  | Training effect | SILS vs Multiport lap general tasks | Eye | No | NASA-TLX, Borg | Yes | Distasi 2017 ^12^ |
|  | Expertise | Laparoscopic suturing | Cardiac | No | SURG-TLX | Yes | Modi 2018 ^6^ |
|  | Expertise | Laparoscopic suturing | fNIRS | Yes | SURG-TLX | Yes | Modi 2018 ^6^ |
|  | Training effect | General Robotic tasks | Eye | Yes | NASA-TLX | Yes | Wu 2020 ^59^ |
|  | Hand dominancy | Sim dissection with dominant vs non dominant hand | Eye | Yes | - | - | Dalveren G 2017 ^60^ |
| Intrinsic Load + Germane | Task difficulty + Expertise | General Lap tasks | EEG | Yes | - | - | Maddox 2015 ^61^ |
| Intrinsic Load + Germane | Task difficulty + Expertise | Simulated Tympanoplasty and stapedectomy | Eye | Yes | NASA-TLX | Yes | Anschueutz 2019 ^62^ |
| Intrinsic Load + Germane | Task difficulty + Expertise | General Lap tasks | Eye | Yes | 7-point likert scale of mental concentration and mental stress | Yes | Berguer 2001 ^63^ |
| Intrinsic Load + Germane | Task difficulty + Hand dominance | General Lap tasks | Eye | Yes | - | - | Dalveren G 2018 (1+2) ^64,65^ |
| Intrinsic Load + Germane | Task difficulty + Training effect | Laparoscopic suturing | fNIRS | Yes | - | - | Izzetoglu 2021 ^66^ |
| Germane Load + Extraneous | Expertise + Ambient noise | Sim Lap Appendicectomy | Eye | Yes | NASA-TLX | Yes | Gao 2018 ^45^ |
| Intrinsic + Extraneous + Germane | Different tasks + auditory noise + training effect | General Lap tasks | Cardiac | Yes | NASA-TLX | Yes | Zakeri 2020 ^67^ |
| Intrinsic + Extraneous + Germane | Different tasks + auditory noise + training effect | General Lap tasks | Eye | No | NASA-TLX | Yes | Zakeri 2020 ^67^ |
| Uncontrolled | Real life operation | Perfusionists performing CPB | Cardiac | Yes | SURG-TLX | used for correlation | Dias 2023 ^68^ |
|  | Real life operation | RATs vs Open (Thoracic Surgery) | Cardiac | Yes | NASA-TLX | Yes | Mazella 2023 ^69^ |
|  | Real life operations | Whole operating day | Cardiac | No | NASA-TLX | Yes | Rieger 2014 ^70^R |

CLT: Cognitive Load Theory; CWL: Cognitive Workload; NASA-TLX: National Aeronautics Space Administration Task Load Index; SURG-TLx: Surgery Task Load Index; CABG: Coronary Artery Bypass Graft; HR: Heart rate; SILC: Single Incision Laparoscopic Surgery; CLC: Conventional Laparoscopic Cholecystectomy; RC: Robotic Cholecystectomy; SILS: Single Incision Laparoscopic Suturing; 2D: Two Dimensions; 3D: Three Dimensions; Lap: Laparoscopic; Sim: Simulated; TEVAR: Thoracic Endovascular Aortic Repair; CPB: CardioPulmonary Bypass; RATS: Robotic-Assisted Thoracic Surgery

**Supplemental Table 12** Sensitivity and specificity of comparing single modality with multimodal studies, and the sensitivity of the different metrics which were reported in three or more experiments.

| **Modality/**  **Metric** | **N= controlled experiments** | **True positives** | **False positives** | **Sensitivity (TP/(TP+FN))** | **True negatives** | **False negatives** | **Specificity(TN/(FP+TN))** |
| --- | --- | --- | --- | --- | --- | --- | --- |
| Cardiac | 23 | 15 | 1 | 71.43% | 1 | 6 | 50.00% |
| With Multimodal | 23 | 18 | 1 | 85.71% | 1 | 3 | 50.00% |
| fNIRS | 11 | 8 | 1 | 80.00% | 0 | 2 | N/A |
| With Multimodal | 11 | 7 | 1 | 87.50% | 0 | 1 | N/A |
| EEG | 15 | 14 | 1 | 100.00% | 0 | 0 | N/A |
| With Multimodal | No change | | | | | | |
| Ocular | 33 | 27 | 1 | 90.00% | 2 | 3 | 66.67% |
| With Multimodal | 33 | 26 | 1 | 92.86% | 2 | 2 | 66.67% |
| Only Multimodal experiments | 13 | 10 | 0 | 76.92% | 0 | 3 | N/A |
|  | N= controlled experiments | True positives | False positives | Sensitivity (TP/(TP+FN)) | True negatives | False negatives |  |
| Cardiac | 19 | 14 | 1 | 82.35% | 1 | 3 |  |
|  |  |  |  |  |  |  |  |
| Mean HR | 15 | 9 | 0 | 69.23% | 2 | 4 |  |
| LF/HF | 7 | 3 | 1 | 50.00% | 0 | 3 |  |
| SDNN | 4 | 3 | 0 | 100.00% | 1 | 0 |  |
| RMSSD | 3 | 1 | 1 | 50.00% | 0 | 1 |  |
| PNN50 | 3 | 2 | 1 | 100.00% | 0 | 0 |  |
| Max HR | 3 | 2 | 0 | 66.67% | 0 | 1 |  |
| Median HR | 3 | 1 | 0 | 33.33% | 0 | 2 |  |
| fNIRS | 11 | 8 | 1 | 80.00% | 0 | 2 |  |
| EEG | 15 | 14 | 1 | 100.00% | 0 | 0 |  |
| Alpha | 3 | 2 | 0 | 66.67% | 0 | 1 |  |
| Theta | 3 | 1 | 1 | 50.00% | 0 | 1 |  |
| Delta | 2 | 2 | 0 | 100.00% | 0 | 0 |  |
| Beta | 3 | 2 | 1 | 100.00% | 0 | 0 |  |
| Ocular | 33 | 27 | 1 | 90.00% | 2 | 3 |  |
| Pupil Size | 13 | 10 | 0 | 76.92% | 0 | 3 |  |
| Change in Pupil Diameter | 9 | 7 | 0 | 87.50% | 1 | 1 |  |
| Fixation Number | 8 | 5 | 0 | 71.43% | 0 | 2 |  |
| Fixation Duration | 8 | 3 | 0 | 37.50% | 0 | 5 |  |
| Blink Rate | 8 | 5 | 0 | 62.50% | 0 | 3 |  |
| Gaze Entropy | 5 | 5 | 0 | 100.00% | 0 | 0 |  |
| Gaze Velocity | 5 | 5 | 0 | 100.00% | 0 | 0 |  |
| Fixation Frequency | 5 | 3 | 1 | 75.00% | 0 | 1 |  |
| Saccade Number | 4 | 3 | 1 | 100.00% | 0 | 0 |  |
| Pupil Entropy | 3 | 1 | 1 | 50.00% | 0 | 1 |  |
| Saccade Duration | 3 | 1 | 1 | 50.00% | 0 | 1 |  |
| Dwell Time | 3 | 2 | 1 | 100.00% | 0 | 0 |  |
| Saccade Velocity | 3 | 1 | 0 | 33.33% | 0 | 2 |  |

Abbreviations: TP, True Positive; TN, True Negative; FP, False Positive; FN, False Negative.

Calculation of specificity for fNIRS and EEG metrics was not possible due to a lack of true negative experiments (experiments whereby the subjective and objective modality reported no change in CWL against the task paradigm). Each CLT domain investigated was counted as a separate experiment, and individual metric sensitivity was reported if used in three studies or more; uncontrolled studies were excluded.

**References:**

1. Galoyan T, Betts K, Abramian H, Reddy P, Izzetoglu K, Shewokis PA. Examining Mental Workload in a Spatial Navigation Transfer Game via Functional near Infrared Spectroscopy. *Brain Sci*. Jan 4 2021;11(1)doi:10.3390/brainsci11010045

2. Sweller J. Cognitive load theory, learning difficulty, and instructional design. *Learning and Instruction*. 1994/01/01/ 1994;4(4):295-312. doi:10.1016/0959-4752(94)90003-5

3. Kennedy-Metz LR, Arshanskiy M, Keller S, Arney D, Dias RD, Zenati MA. Association Between Operating Room Noise and Team Cognitive Workload in Cardiac Surgery. *IEEE Conf Cogn Comput Asp Situat Manag*. Jun 2022;2022:89-93. doi:10.1109/cogsima54611.2022.9830675

4. Z P, KE W, S M, et al. Higher operating theatre temperature during burn surgery increases physiological heat strain, subjective workload, and fatigue of surgical staff. *PloS one*. 06/02/2023 2023;18(6)doi:10.1371/journal.pone.0286746

5. Modi HN, Singh H, Darzi A, Leff DR. Multitasking and Time Pressure in the Operating Room: Impact on Surgeons' Brain Function. *Ann Surg*. Oct 2020;272(4):648-657. doi:10.1097/SLA.0000000000004208

6. Modi HN, Singh H, Orihuela-Espina F, et al. Temporal Stress in the Operating Room: Brain Engagement Promotes "Coping" and Disengagement Prompts "Choking". *Ann Surg*. Apr 2018;267(4):683-691. doi:10.1097/SLA.0000000000002289

7. Modi HN, Singh H, Fiorentino F, et al. Association of Residents' Neural Signatures With Stress Resilience During Surgery. *JAMA Surg*. Oct 1 2019;154(10):e192552. doi:10.1001/jamasurg.2019.2552

8. Singh H, Modi HN, Ranjan S, et al. Robotic Surgery Improves Technical Performance and Enhances Prefrontal Activation During High Temporal Demand. *Ann Biomed Eng*. Oct 2018;46(10):1621-1636. doi:10.1007/s10439-018-2049-z

9. Abdelrahman AM, Bingener J, Yu D, et al. Impact of single-incision laparoscopic cholecystectomy (SILC) versus conventional laparoscopic cholecystectomy (CLC) procedures on surgeon stress and workload: a randomized controlled trial. *Surg Endosc*. Mar 2016;30(3):1205-11. doi:10.1007/s00464-015-4332-5

10. Heemskerk J, Zandbergen HR, Keet SW, et al. Relax, it's just laparoscopy! A prospective randomized trial on heart rate variability of the surgeon in robot-assisted versus conventional laparoscopic cholecystectomy. *Dig Surg*. 2014;31(3):225-32. doi:10.1159/000365580

11. Morales JM, Ruiz-Rabelo JF, Diaz-Piedra C, Di Stasi LL. Detecting Mental Workload in Surgical Teams Using a Wearable Single-Channel Electroencephalographic Device. *J Surg Educ*. Jul-Aug 2019;76(4):1107-1115. doi:10.1016/j.jsurg.2019.01.005

12. Di Stasi LL, Diaz-Piedra C, Ruiz-Rabelo JF, Rieiro H, Sanchez Carrion JM, Catena A. Quantifying the cognitive cost of laparo-endoscopic single-site surgeries: Gaze-based indices. *Appl Ergon*. Nov 2017;65:168-174. doi:10.1016/j.apergo.2017.06.008

13. Kelkar A, Kelkar J, Chougule Y, Bolisetty M, Singhvi P. Cognitive workload, complications and visual outcomes of phacoemulsification cataract surgery: Three-dimensional versus conventional microscope. *Eur J Ophthalmol*. Sep 2022;32(5):2935-2941. doi:10.1177/11206721211062034

14. Zhang JY, Shen ZH, Wang BP, Liu F, Li J. Influence of 3D laparoscopic surgery on surgeon's visual pattern and mental workload. *J Med Eng Technol*. Jul 2021;45(5):375-379. doi:10.1080/03091902.2021.1907466

15. Shugaba A, Subar DA, Slade K, et al. Surgical stress: the muscle and cognitive demands of robotic and laparoscopic surgery. *Annals of surgery open: perspectives of surgical history, education, and clinical approaches*. 2023;4(2)

16. Dedmon MM, O'Connell BP, Yawn RJ, et al. Measuring Mental Stress During Otologic Surgery Using Heart Rate Variability Analysis. *Otol Neurotol*. Apr 2019;40(4):529-534. doi:10.1097/MAO.0000000000002187

17. Martin J, Schneider F, Kowalewskij A, et al. Linear and non-linear heart rate metrics for the assessment of anaesthetists' workload during general anaesthesia. *Br J Anaesth*. Dec 2016;117(6):767-774. doi:10.1093/bja/aew342

18. Schulz CM, Schneider E, Fritz L, et al. Eye tracking for assessment of workload: a pilot study in an anaesthesia simulator environment. *Br J Anaesth*. Jan 2011;106(1):44-50. doi:10.1093/bja/aeq307

19. Pimentel G, Rodrigues S, Silva PA, Vilarinho A, Vaz R, Silva Cunha JP. A wearable approach for intraoperative physiological stress monitoring of multiple cooperative surgeons. *Int J Med Inform*. Sep 2019;129:60-68. doi:10.1016/j.ijmedinf.2019.05.028

20. Song MH, Tokuda Y, Nakayama T, Sato M, Hattori K. Intraoperative heart rate variability of a cardiac surgeon himself in coronary artery bypass grafting surgery. *Interact Cardiovasc Thorac Surg*. Jun 2009;8(6):639-41. doi:10.1510/icvts.2008.195941

21. Guru KA, Shafiei SB, Khan A, Hussein AA, Sharif M, Esfahani ET. Understanding Cognitive Performance During Robot-Assisted Surgery. *Urology*. Oct 2015;86(4):751-7. doi:10.1016/j.urology.2015.07.028

22. Hussein AA, Shafiei SB, Sharif M, et al. Technical mentorship during robot-assisted surgery: a cognitive analysis. *BJU Int*. Sep 2016;118(3):429-36. doi:10.1111/bju.13445

23. Plazak J, DiGiovanni DA, Collins DL, Kersten-Oertel M. Cognitive load associations when utilizing auditory display within image-guided neurosurgery. *Int J Comput Assist Radiol Surg*. Aug 2019;14(8):1431-1438. doi:10.1007/s11548-019-01970-w

24. Shafiei SB, Hussein AA, Muldoon SF, Guru KA. Functional Brain States Measure Mentor-Trainee Trust during Robot-Assisted Surgery. *Sci Rep*. Feb 26 2018;8(1):3667. doi:10.1038/s41598-018-22025-1

25. Yang J, Barragan JA, Farrow JM, Sundaram CP, Wachs JP, Yu D. An Adaptive Human-Robotic Interaction Architecture for Augmenting Surgery Performance Using Real-Time Workload Sensing—Demonstration of a Semi-autonomous Suction Tool. *Human Factors*. 2022:00187208221129940.

26. Diaz-Piedra C, Sanchez-Carrion JM, Rieiro H, Di Stasi LL. Gaze-based Technology as a Tool for Surgical Skills Assessment and Training in Urology. *Urology*. Sep 2017;107:26-30. doi:10.1016/j.urology.2017.06.030

27. Di Stasi LL, Diaz-Piedra C, Rieiro H, et al. Gaze entropy reflects surgical task load. *Surg Endosc*. Nov 2016;30(11):5034-5043. doi:10.1007/s00464-016-4851-8

28. Jiang X, Zheng B, Tien G, Atkins MS. Pupil response to precision in surgical task execution. *Medicine Meets Virtual Reality 20*. IOS Press; 2013:210-214.

29. Jiang X, Atkins MS, Tien G, Zheng B, Bednarik R. Pupil dilations during target-pointing respect Fitts' Law. 2014:175-182.

30. Zhang J, Liu S, Feng Q, et al. Ergonomic assessment of the mental workload confronted by surgeons during laparoscopic surgery. *The American Surgeon*. 2018;84(9):1538-1543.

31. Zheng B, Jiang X, Atkins MS. Detection of changes in surgical difficulty: evidence from pupil responses. *Surgical innovation*. 2015;22(6):629-635.

32. Guru KA, Esfahani ET, Raza SJ, et al. Cognitive skills assessment during robot-assisted surgery: separating the wheat from the chaff. *BJU Int*. Jan 2015;115(1):166-74. doi:10.1111/bju.12657

33. Weinger MB, Reddy SB, Slagle JM. Multiple measures of anesthesia workload during teaching and nonteaching cases. *Anesth Analg*. May 2004;98(5):1419-25, table of contents. doi:10.1213/01.ane.0000106838.66901.d2

34. Bakhsh A, Martin GFJ, Bicknell CD, Pettengell C, Riga C. An Evaluation of the Impact of High-Fidelity Endovascular Simulation on Surgeon Stress and Technical Performance. *J Surg Educ*. May-Jun 2019;76(3):864-871. doi:10.1016/j.jsurg.2018.10.015

35. Carnevali L, Bignami E, Gambetta S, et al. Cardiac autonomic and cortisol stress responses to real operations in surgeons: relationship with individual psychobiological characteristics and experience. OriginalPaper. *BioPsychoSocial Medicine*. 2023-02-21 2023;17(1):1-14. doi:doi:10.1186/s13030-023-00266-5

36. D H, M S, V S, et al. Differences in beginner and expert neurointerventionalists" heart rate variability during simulated neuroangiographies. *Interventional neuroradiology : journal of peritherapeutic neuroradiology, surgical procedures and related neurosciences*. 09/19/2022 2022;doi:10.1177/15910199221128439

37. Schulz CM, Schneider E, Kohlbecher S, et al. The influence of anaesthetists' experience on workload, performance and visual attention during simulated critical incidents. *J Clin Monit Comput*. Oct 2014;28(5):475-80. doi:10.1007/s10877-013-9443-8

38. A N, MA Y, U K, et al. Assessing bimanual motor skills with optical neuroimaging. *Science advances*. 10/03/2018 2018;4(10)doi:10.1126/sciadv.aat3807

39. Walia P, Fu Y, Norfleet J, et al. Error-related brain state analysis using electroencephalography in conjunction with functional near-infrared spectroscopy during a complex surgical motor task. *Brain Informatics*. 2022;9(1):1-25.

40. Bednarik R, Bartczak P, Vrzakova H, et al. Pupil size as an indicator of visual-motor workload and expertise in microsurgical training tasks. 2018:1-5.

41. Berges AJ, Vedula SS, Chara A, Hager GD, Ishii M, Malpani A. Eye tracking and motion data predict endoscopic sinus surgery skill. *The Laryngoscope*. 2023;133(3):500-505.

42. Cai B, Xu N, Duan S, et al. Eye tracking metrics of orthopedic surgeons with different competency levels who practice simulation-based hip arthroscopic procedures. *Heliyon*. 2022;8(12)

43. Cao Y, Kobayashi Y, Zhang B, Liu Q, Sugano S, Fujie MG. Evaluating proficiency on a laparoscopic suturing task through pupil size. IEEE; 2015:677-681.

44. Erridge S, Ashraf H, Purkayastha S, Darzi A, Sodergren MH. Comparison of gaze behaviour of trainee and experienced surgeons during laparoscopic gastric bypass. *Br J Surg*. Feb 2018;105(3):287-294. doi:10.1002/bjs.10672

45. Gao J, Liu S, Feng Q, et al. Quantitative Evaluations of the Effects of Noise on Mental Workloads Based on Pupil Dilation during Laparoscopic Surgery. *Am Surg*. Dec 1 2018;84(12):1951-1956.

46. Gunawardena N, Matscheko M, Anzengruber B, et al. Assessing surgeons' skill level in laparoscopic cholecystectomy using eye metrics. 2019:1-8.

47. Koskinen J, Bednarik R, Vrzakova H, Elomaa A-P. Combined gaze metrics as stress-sensitive indicators of microsurgical proficiency. *Surgical Innovation*. 2020;27(6):614-622.

48. Nguyen JH, Chen J, Marshall SP, et al. Using objective robotic automated performance metrics and task-evoked pupillary response to distinguish surgeon expertise. *World J Urol*. Jul 2020;38(7):1599-1605. doi:10.1007/s00345-019-02881-w

49. Richstone L, Schwartz MJ, Seideman C, Cadeddu J, Marshall S, Kavoussi LR. Eye metrics as an objective assessment of surgical skill. *Ann Surg*. Jul 2010;252(1):177-82. doi:10.1097/SLA.0b013e3181e464fb

50. Tien T, Pucher PH, Sodergren MH, Sriskandarajah K, Yang G-Z, Darzi A. Differences in gaze behaviour of expert and junior surgeons performing open inguinal hernia repair. *Surgical endoscopy*. 2015;29:405-413.

51. Zheng B, Jiang X, Tien G, Meneghetti A, Panton ON, Atkins MS. Workload assessment of surgeons: correlation between NASA TLX and blinks. *Surg Endosc*. Oct 2012;26(10):2746-50. doi:10.1007/s00464-012-2268-6

52. Leff DR, Elwell CE, Orihuela-Espina F, et al. Changes in prefrontal cortical behaviour depend upon familiarity on a bimanual co-ordination task: an fNIRS study. *Neuroimage*. Jan 15 2008;39(2):805-13. doi:10.1016/j.neuroimage.2007.09.032

53. Yu P, Pan J, Wang Z, et al. Quantitative influence and performance analysis of virtual reality laparoscopic surgical training system. *BMC Med Educ*. Feb 10 2022;22(1):92. doi:10.1186/s12909-022-03150-y

54. Crewther BT, Shetty K, Jarchi D, et al. Skill acquisition and stress adaptations following laparoscopic surgery training and detraining in novice surgeons. *Surg Endosc*. Jul 2016;30(7):2961-8. doi:10.1007/s00464-015-4584-0

55. Maimon NB, Bez M, Drobot D, et al. Continuous Monitoring of Mental Load During Virtual Simulator Training for Laparoscopic Surgery Reflects Laparoscopic Dexterity: A Comparative Study Using a Novel Wireless Device. *Front Neurosci*. 2021;15:694010. doi:10.3389/fnins.2021.694010

56. Suarez-Revelo JX, Ochoa-Gomez JF, Hernandez-Valdivieso AM. Neurophysiological changes associated with training in laparoscopic surgery using EEG: a pilot study(). *Annu Int Conf IEEE Eng Med Biol Soc*. Jul 2019;2019:4572-4575. doi:10.1109/EMBC.2019.8856980

57. Wu C, Cha J, Sulek J, et al. Sensor-based indicators of performance changes between sessions during robotic surgery training. *Appl Ergon*. Jan 2021;90:103251. doi:10.1016/j.apergo.2020.103251

58. Dilley J, Singh H, Pratt P, Omar I, Darzi A, Mayer E. Visual behaviour in robotic surgery—demonstrating the validity of the simulated environment. *The International Journal of Medical Robotics and Computer Assisted Surgery*. 2020;16(2):e2075.

59. Wu C, Cha J, Sulek J, et al. Eye-Tracking Metrics Predict Perceived Workload in Robotic Surgical Skills Training. *Hum Factors*. Dec 2020;62(8):1365-1386. doi:10.1177/0018720819874544

60. Dalveren GGM, Çağıltay NE, Özçelik E, Maraş H. Simulation-based environments for surgical practice. IEEE; 2017:1153-1156.

61. Maddox MM, Lopez A, Mandava SH, et al. Electroencephalographic Monitoring of Brain Wave Activity During Laparoscopic Surgical Simulation to Measure Surgeon Concentration and Stress: Can the Student Become the Master? *J Endourol*. Dec 2015;29(12):1329-33. doi:10.1089/end.2015.0239

62. Anschuetz L, Niederhauser L, Wimmer W, et al. Comparison of 3- vs 2-Dimensional Endoscopy Using Eye Tracking and Assessment of Cognitive Load Among Surgeons Performing Endoscopic Ear Surgery. *JAMA Otolaryngol Head Neck Surg*. Sep 1 2019;145(9):838-845. doi:10.1001/jamaoto.2019.1765

63. Berguer R, Smith WD, Chung YH. Performing laparoscopic surgery is significantly more stressful for the surgeon than open surgery. *Surg Endosc*. Oct 2001;15(10):1204-7. doi:10.1007/s004640080030

64. Dalveren GGM, Cagiltay NE. Using eye-movement events to determine the mental workload of surgical residents. *Journal of Eye Movement Research*. 2018;11(4)

65. Menekse Dalveren GG, Cagiltay NE. Insights from surgeons’ eye-movement data in a virtual simulation surgical training environment: effect of experience level and hand conditions. *Behaviour & Information Technology*. 2018;37(5):517-537.

66. Izzetoglu K, Aksoy ME, Agrali A, Kitapcioglu D, Gungor M, Simsek A. Studying Brain Activation during Skill Acquisition via Robot-Assisted Surgery Training. *Brain Sci*. Jul 16 2021;11(7)doi:10.3390/brainsci11070937

67. Zakeri Z, Mansfield N, Sunderland C, Omurtag A. Physiological correlates of cognitive load in laparoscopic surgery. *Sci Rep*. Jul 31 2020;10(1):12927. doi:10.1038/s41598-020-69553-3

68. Dias RD, Kennedy-Metz LR, Srey R, et al. Using Digital Biomarkers for Objective Assessment of Perfusionists’ Workload and Acute Stress During Cardiac Surgery. Springer; 2023:443-454.

69. A M, M C, D G, et al. How Much Stress Does a Surgeon Endure? The Effects of the Robotic Approach on the Autonomic Nervous System of a Surgeon in the Modern Era of Thoracic Surgery. *Cancers*. 02/14/2023 2023;15(4)doi:10.3390/cancers15041207

70. Rieger A, Fenger S, Neubert S, Weippert M, Kreuzfeld S, Stoll R. Psychophysical workload in the operating room: primary surgeon versus assistant. *Surg Endosc*. Jul 2015;29(7):1990-8. doi:10.1007/s00464-014-3899-6
